# Stable isotope ecology of a hyper-diverse community of scincid lizards from arid Australia

**DOI:** 10.1371/journal.pone.0172879

**Published:** 2017-02-28

**Authors:** Maggie R. Grundler, Eric R. Pianka, Nicolás Pelegrin, Mark A. Cowan, Daniel L. Rabosky

**Affiliations:** 1 Museum of Zoology and Department of Ecology and Evolutionary Biology, University of Michigan, Ann Arbor, Michigan, United States of America; 2 Department of Integrative Biology C0930, University of Texas at Austin, One University Station, Austin, Texas, United States of America; 3 Laboratorio de Ecología y Conservación de la Herpetofauna, Instituto de Diversidad y Ecología Animal, (IDEA, CONICET-UNC), and Centro de Zoología Aplicada (UNC), Córdoba, Argentina; 4 Department of Parks and Wildlife, Wanneroo, Western Australia, Australia; Stockholm University, SWEDEN

## Abstract

We assessed the utility of stable isotope analysis as a tool for understanding community ecological structure in a species-rich clade of scincid lizards from one of the world's most diverse lizard communities. Using a phylogenetic comparative framework, we tested whether δ^15^N and δ^13^C isotopic composition from individual lizards was correlated with species-specific estimates of diet and habitat use. We find that species are highly divergent in isotopic composition with significant correlations to habitat use, but this relationship shows no phylogenetic signal. Isotopic composition corresponds to empirical observations of diet for some species but much variation remains unexplained. We demonstrate the importance of using a multianalytical approach to questions of long-term dietary preference, and suggest that the use of stable isotopes in combination with stomach content analysis and empirical data on habitat use can potentially reveal patterns in ecological traits at finer scales with important implications for community structuring.

## Introduction

The trophic ecology of vertebrate communities is contingent on an array of both historical and contemporary interactions. By characterizing ecological traits among species and their relation to phylogenetic and trophic structure, researchers can reveal the processes that influence community composition in diverse species assemblages [1–3]. However, detailed profiles of diet can be difficult to obtain and may only provide a “snapshot” view in time (Dalerum and Angerbjörn 2005; Inger *et al*. 2006; Lugendo *et al*. 2006; Cherel *et al*. 2007). For studying trophic interactions in vertebrate communities, stable isotope analysis can potentially provide integrated measures of resource use through time, without requiring intensive sampling over broad temporal and spatial scales [4–5]. Stable isotope profiles thus afford complementary insights to analysis of stomach contents and resulting estimates of community trophic structure.

These analyses are relatively robust to variability through time because an organism’s tissues carry a unique isotopic signature based on ecophysiological properties and the sources from which nutrients are assimilated, providing a proxy that can be used for the analysis of a species’ trophic niche [4, 6–8]. This is a result of the relatively minimal fractionation of the stable isotope of carbon and the predictable enrichment of the stable isotope of nitrogen during trophic transfer, resulting in the preservation of isotopic signature of the primary sources of dietary carbon and reliable measures of trophic position [5].

A variety of existing studies have compared isotopic signatures to multivariate ecological data and phylogenies to answer questions about community assembly ranging from niche space occupancy to trait differentiation in ecomorphological radiations [5, 9–13]. While many advances in isotope ecology have involved marine food webs, fewer studies have combined isotope ratios with both ecological and phylogenetic data in terrestrial community assemblages; those that do focus heavily on birds and mammals, with little data for reptile or amphibian communities [14–17]. However, isotopic signatures of squamate reptiles are expected to capture temporally-averaged patterns of trophic resource use, due to the slow turnover rates of δ^15^N and δ^13^C isotopes in tissues of poikilothermic vertebrates [15–16].

In this study, we combine stable isotope analysis with long-term ecological monitoring data and a community phylogeny to examine whether isotope ratios provide information about the trophic ecology of a diverse clade of scincid lizards from the spinifex deserts of the western Australian arid zone. This clade—the sphenomorphine skinks [18]—includes the genera *Lerista* and *Ctenotus* and is well-known as Australia's most diverse terrestrial vertebrate radiation [19]. Closely-related species within these and other genera are divergent in both habitat use and dietary resources [1,20]. We first asked whether species are distinguishable in isotopic space, and determined if there is phylogenetic signal for isotopic composition. We then investigated whether differences in isotopic composition were predicted by dietary differences as revealed by detailed stomach content analyses. We also tested whether species whose diets are dominated by prey from higher trophic levels (e.g., secondary consumers, such as spiders) would demonstrate higher δ^15^N values. Finally, we examined whether the variance in isotopic composition among species was related to patterns of habitat use. We predicted that species with similar isotopic signatures would show divergent habitat use patterns, because competition for dietary resources may have mediated shifts in habitat use in arid Australian lizards [1, 21–25].

## Materials and methods

### Isotopic analysis

We quantified isotopic composition from ethanol-preserved liver tissue from adult sphenomorphine skinks of three genera (*Ctenotus*, *Lerista*, and *Eremiascincus)* that regionally co-occur in the western Australian arid zone. Whole-animal voucher specimens and tissue samples were collected from populations at Lorna Glen (Matuwa) and Yamarna Station in Western Australia’s Great Victoria Desert in 2004–2006 under permit SF0004654 to DLR (Western Australia Department of Parks and Wildlife). These locations are approximately 321.5 km apart but are broadly similar in physiography and have similar squamate reptile communities. For continuity with previous literature [1,24,26], we note that one of the focal species found in both regions, *Ctenotus helenae*, was recently synonymized with *Ctenotus inornatus* [27] and the current name is used here. A total of 141 individuals from 14 species were analyzed for isotopic content, with 2–20 individuals sampled per species. Liver tissue preserved in EtOH was dried and ground using a 1/8” metal bead lysing matrix from MP Biomedicals. Pulverized tissue was weighed in tin capsules and stored in a desiccator until used for analysis. Carbon and nitrogen stable isotope ratios (δ^13^C and δ^15^N respectively) were measured on a Finnigan MAT Delta Plus IRMS coupled to an elemental analyzer (Carlo Erba NC2500) at the Cornell University Stable Isotope Laboratory. Stable isotope ratios were expressed as a delta (δ) in ‰ (per mil or parts per thousand) according to:

$$\left[\frac{\left(R_{\text{sample}}-R_{\text{standard}}\right)}{R_{\text{standard}}}\right]\times 1000=d_{\text{sample-standard}}$$

where *R*_sample_ is the ratio of the heavy isotope to light isotope (^13^C/^12^C or ^15^N/^14^N) in the sample, *R*_standard_ is the ratio of the heavy isotope to light isotope in the working reference gas, which is calibrated against an internationally known IAEA standard (V-PDB for δ^13^C and atmospheric nitrogen for δ^15^N), and *d*_sample-standard_ is the difference in isotopic composition of the sample relative to that of the reference, expressed in units per mil (‰). Errors associated with linearity were corrected at the Cornell Laboratory using a two-point normalization (linear regression) of all δ^13^C and δ^15^N data using two additional in-house standards that loosely resemble the samples being analyzed (HCRN, a corn standard, and CBT, a trout standard; these standards are run once every ten samples to identify variability in measurement or long-term drift). Note that preservation in EtOH has not been found to have a significant effect on isotopic composition of tissue from a range of consumer taxa [28–30], although one study has found that for liver tissue specifically, preservation in 95% EtOH leads to an increase in δ^13^C of around 1.5 ‰ [31].

### Ecological data

Data on habitat use was collected by DLR and collaborators [1] as a total of 14 habitat variables relating to vegetation structure and substrate type, collected at Lorna Glen for each of 928 individual pitfall traps as part of a long term study on community structure for arid-zone vertebrates. Examples of measurements include a visual estimate, within a 3-m radius of each trap, of the percentage of ground covered by vegetation such as hummock grasses (spinifex) or chenopod shrubs; the percentage of exposed ground composed of substrates such as sand or gravel; and additional measurements of habitat qualities such as soil compaction and volume of woody debris. The mean value was computed for each habitat variable for each species over all pitfall traps where the species was collected, and means were transformed according to the method (log, logit, or arcsine square root) leading to the best approximation of normality. A complete description of habitat variables and methodology used to derive species-specific habitat scores can be found in [1]. Habitat data are available for 9 species in the present study, all of which are represented in the Lorna Glen isotope dataset and 7 of which are represented in the Yamarna isotope dataset.

Skinks were collected at Yamarna by ERP and collaborators from 1978–1979 and deposited in collections at the Western Australian Museum and the University of Texas. Stomach contents were removed from preserved animals through dissection, and prey items were sorted into 63 categories, including but not limited to centipedes, spiders, termites, ants, and beetles. Percent stomach volume occupied by each prey class was calculated for individual lizards, and means were calculated for each species. Ontogenetic shifts in diet are generally considered rare in small lizards [32–34] and fewer than five percent of lizards in our dataset had body sizes consistent with juvenile status. Hence, we believe ontogenetic dietary change is only a marginal contributor to the overall variation in isotopic composition within the focal taxa. Empirical dietary data were available for only 8 species after selection for sample size (N ≥ 5), and subsequent statistical analyses were conducted separately for Lorna Glen (6 of 8 species represented) and Yamarna (8 of 8 species represented) communities.

### Data analysis

All statistical and phylogenetic analyses were performed in the R programming/statistical environment [35]. We first assessed variation in isotopic composition among species using a oneway ANOVA with unequal variances for δ^13^C and δ^15^N values separately (δ^13^C: Bartlett’s K-squared = 33.39, d.f. = 16, *P* = 0.007; δ^15^N: Bartlett’s K-squared = 32.68, d.f. = 16, *P* = 0.008). A oneway ANOVA was also performed for δ^13^C and δ^15^N values to determine if there was any effect of geographic region (Yamarna vs. Lorna Glen) on isotopic composition.

We tested for phylogenetic signal for δ^13^C and δ^15^N values pooled from both communities using a branch-length transformation test (Pagel's λ; [36]) as implemented in the function phylosig from the package phytools [37]. Briefly, the method compares the likelihood of a phenotypic dataset under a model where patterns of trait covariation are predicted by phylogeny to a null model where no phylogenetic correlation in species trait values exists. Analyses were conducted on the maximum clade credibility tree from a previously-published distribution of time-calibrated species-level phylogenies for Australian sphenomorphine skinks that included all species from the Lorna Glen and Yamarna communities [19]. We also tested for phylogenetic signal for δ^13^C and δ^15^N values in each regional community separately, to minimize the potential effects of regional variation on species mean values.

We tested for an association between isotopic composition and habitat use using a Mantel test to compare pairwise Euclidean distance matrices of species’ mean values for δ^13^C and δ^15^N to a distance matrix computed from fourteen habitat variables from [1]. Carbon and nitrogen values were analyzed together and separately, factored by location. To assess the relationship between dietary similarity and stable isotope composition, we used a Mantel test to compare species' isotopic means to dietary similarity as inferred from stomach content analyses. Because the majority of species from these datasets belong to the genus *Ctenotus*, we also conducted these analyses with the non-*Ctenotus* species removed. Non-*Ctenotus* taxa included several species from the genus *Lerista*, a fossorial and highly limb reduced clade that is ecologically distinct from sphenomorphine lineages. We also tested for an association between stomach contents and habitat use to identify correlations between trophic ecology and microhabitat that may have gone undetected by isotopic analysis. We used Mantel tests implemented in the ade4 package [38].

We also conducted phylogenetically informed three-way Mantel tests for habitat and diet associations with isotopic composition separately for the two regional communities using a permutation algorithm, demonstrated to be less prone to type-I error than the traditional Mantel test [39–40].

To further compare results of stable isotope analysis with stomach content analyses, we tested whether isotopic measures of trophic position were correlated with the estimated trophic rank of each species as inferred from stomach content analysis. We used species' positions along the δ^15^N axis as a proxy for trophic ordering in isotopic space; these values were compared to species’ ordering predicted by a weighted trophic rank calculated based on the mean proportion of prey items in a species’ stomach and their general trophic position. Prey items were assigned a trophic rank from 1 to 5 under the following coding scheme: 1 = producer; 2 = primary grazer (exclusively phytophagous consumer); 3 = omnivorous consumer (phytophagous and predator of primary grazers); 4 = predator of omnivorous consumers; and 5 = predator of vertebrate prey species. Prey items that were identified only as higher order taxonomic groups with the potential to contain species from multiple ranked categories were assigned a value intermediate between the two ranks. The weighted trophic rank was then calculated as follows:

$$\text{Weighted trophic rank}=\sum_{i=1}^{n}\left(p_{i}T_{i}\right)$$

where *i* is a given prey item, *n* is the number of distinct prey types present in a species’ stomach, *p*_*i*_ is the proportion of the *i*th prey item in the stomach, and *T*_*i*_ is the corresponding trophic rank of the prey item. This calculation ensures that a species’ trophic score will be more strongly affected by the rank of prey items on which it concentrates heavily, similar to the process of isotopic assimilation wherein species’ tissues accumulate the isotopes (and more strongly reflect the trophic level, with predictable isotopic enrichment) of the prey they preferentially consume.

## Results

**Fig 1:**
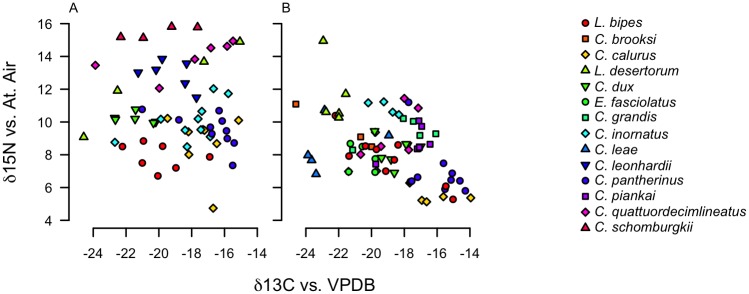
Differentiation in isotopic space for scincid lizards from Lorna Glen and Yamarna communities. δ^13^C and δ^15^N signatures measured in units per mil for individuals collected from the Lorna Glen (A) and Yamarna (B) regional communities; note clustering of individuals by taxon. Species are significantly differentiated in isotopic space (Table 1), suggesting that stable isotope profiles contain information about species-specific ecological traits.

**Fig 2:**
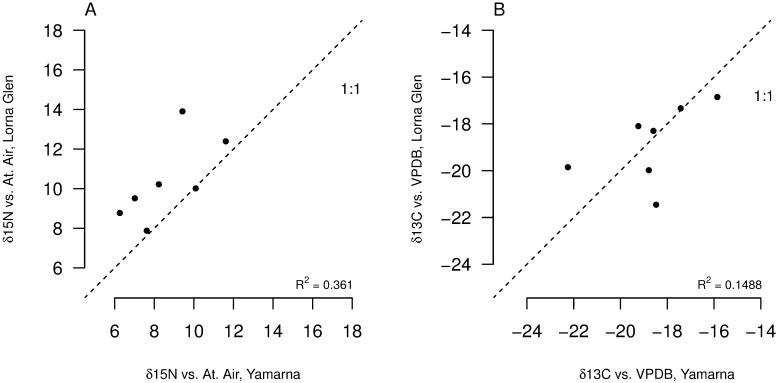
Regional differences in mean δ^13^C and δ^15^N signatures for species present in both communities. (A) Species’ mean δ^15^N from Lorna Glen vs. mean δ^15^N from Yamarna. Signatures tend to be higher for species from Lorna Glen, and there is no significant correlation between communities (*r* = 0.68, *p* = 0.09). 1:1 relationship shown for reference as a dashed line. (B) Species’ mean δ^13^C from Lorna Glen vs. mean δ^13^C from Yamarna. There is no significant correlation between communities (*r* = 0.54, *p* = 0.21), but a near significant correlation emerges upon removal of the outlier point representing *Ctenotus dux* (*r* = 0.80, *p* = 0.057; r^2^ = 0.548). 1:1 relationship shown for reference as a dashed line.

**Fig 3:**
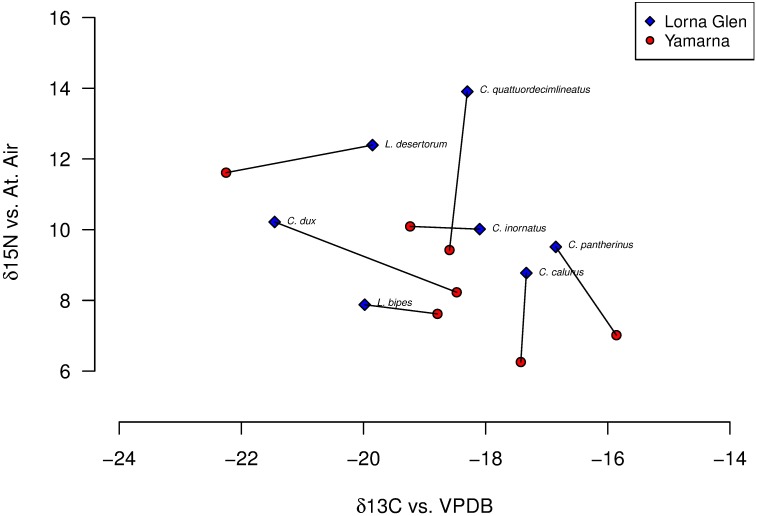
Regional shifts in mean stable isotope signature. Mean δ^15^N and δ^13^C signatures of the same species are significantly different depending on location (*p* = 0.0004 and *p* = 0.0267 respectively). There is no clear trend for the shift in δ^13^C signature, but δ^15^N signatures are lower in Yamarna for all species except *C*. *inornatus*.

**Fig 4:**
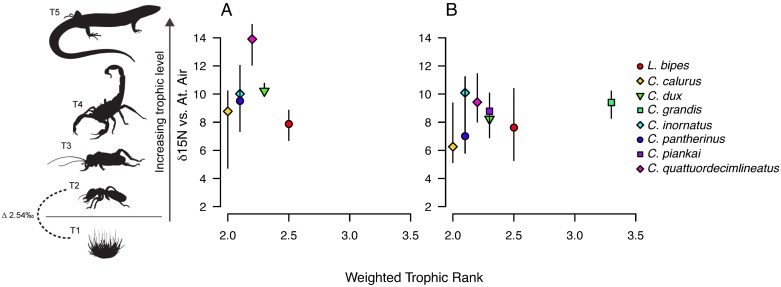
Trophic estimates from stomach contents do not correlate with δ^15^N ranking. (A) Mean δ^15^N signature vs. weighted trophic rank based on stomach content analyses for the Lorna Glen regional community (based on gut contents for species collected in Yamarna). Lines represent δ^15^N range for each species. Isotopic signatures suggest that the focal taxa span 4–5 trophic levels (assumed enrichment 2.5‰ per level), while the corresponding range inferred from stomach contents is 2–3. There is no significant correlation between δ^15^N ranking and estimated trophic rank (*r* = -0.20, *p* = 0.70). (B) Corresponding analysis for the Yamarna regional community. Isotopic estimate of trophic level range is narrower (3) than for Lorna Glen, but more similar to estimates derived from stomach contents (2–3). There is no significant correlation between δ^15^N ranking and estimated trophic rank (*r* = 0.35, *p* = 0.39). Diet data were not available for all species present in the Yamarna dataset, and the full set appears to span 4–5 trophic levels, see Fig 1). Prey item images are numbered by assigned trophic rank (see text for details) and aligned along the y-axis according to estimated relative nitrogen content (as predicted from a 2–3‰ enrichment per trophic transfer [41] beginning at 0‰ for spinifex primary producers; but note *Acacia* vegetation is enriched in δ^15^N, and prey items associated with these plants may reflect this enrichment [42]).

We found a significant effect of species category on both δ^13^C and δ^15^N values (p < 0.0001 for both) (Table 1, Fig 1), indicating that species differ significantly in their position in isotopic space. There was also a significant effect of location on δ^15^N values (p < 0.0001), but not on δ^13^C values (*p* = 0.755) (Table 1). We found that δ^15^N values for several species collected at Yamarna were significantly lower than values for those same species collected at Lorna Glen; the ranges for both isotopes also differed by location (Figs 2 and 3, Tables 1 and 2). Assuming a δ^15^N enrichment of 2.5‰ for each trophic transfer (a meta-analytical study of isotopic enrichment [41] does not include lizards, but finds that tissue from birds yields values of trophic enrichment between 2–3‰, with bird liver specifically yielding measurements closer to 2‰), both communities appear to span four to five trophic levels (Fig 1). This was not consistent with estimates of trophic position from stomach content data (Table 3, Fig 4).

**Table 1:** ANOVA results of stable isotope analysis showing effects of species and location.

	d.f.	δ^15^N		δ^13^C	
		*F*	*P*	*F*	*P*
Species	13	14.1	**<0.0001**	6.994	**<0.0001**
Location	1	36.37	**<0.0001**	0.098	0.755
Species:Location	6	4.521	**0.0004**	2.491	**0.0267**
Species, Yamarna	11	6.313	**<0.0001**	8.937	**<0.0001**
Species, Lorna Glen	8	25.21	**<0.0001**	3.555	**0.00236**

Significant *P*-values are shown in boldface.

**Table 2:** Means and ranges of δ^15^N and δ^13^C signatures for species from Yamarna and Lorna Glen.

Species	Mean δ^15^N	Mean δ^13^C	Range δ^15^N	Range δ^13^C
Yamarna				
*Ctenotus calurus*	6.26	-17.42	4.24	7.45
*Ctenotus dux*	8.23	-18.47	2.54	2.81
*Ctenotus grandis*	9.4	-17.96	1.92	5.07
*Ctenotus inornatus*	10.09	-19.24	4.22	1.87
*Ctenotus leae*	8.47	-22.54	3.90	4.93
*Ctenotus pantherinus*	7.02	-15.86	5.40	3.46
*Ctenotus piankai*	8.78	-17.42	2.64	3.31
*Ctenotus quattuordecimlineatus*	9.42	-18.59	3.42	3.51
*Ctenotus brooksi*	9.56	-21.75	2.60	4.62
*Eremiascincus fasciolatus*	7.77	-20.56	1.74	1.62
*Lerista bipes*	7.62	-18.79	5.11	7.17
*Lerista desertorum*	11.61	-22.25	4.68	1.37
Range	5.35	6.68		
Lorna Glen				
*Ctenotus calurus*	8.77	-17.33	5.48	4.33
*Ctenotus dux*	10.22	-21.46	0.81	2.50
*Ctenotus inornatus*	10.02	-18.10	3.54	6.91
*Ctenotus leonhardii*	12.91	-19.28	2.56	3.55
*Ctenotus pantherinus*	9.51	-16.85	3.42	5.61
*Ctenotus quattuordecimlineatus*	13.09	-18.30	2.88	8.39
*Ctenotus schomburgkii*	15.48	-20.03	0.68	4.68
*Lerista bipes*	7.88	-19.98	2.13	5.32
*Lerista desertorum*	12.39	-19.85	5.82	9.52
Range	7.60	4.61		

Units are measured in parts per thousand (‰, per mil) of international standards.

**Table 3:** Weighted trophic rank of sphenomorphine species based on stomach content analyses.

Species	Major Prey	Proportion of Major Prey in Diet	Trophic Rank of Major Prey	Average Trophic Rank of Other Prey	Weighted Trophic Rank of Sphenomorphine Consumer	Mean δ^15^N of Sphenomorphine Consumer	
						Lorna Glen	Yamarna
*Lerista bipes*	Isoptera	0.48	2	3.1	2.5	7.88	7.62
*Ctenotus calurus*	Isoptera	0.14	2	2.1	2.0	8.77	6.26
*Ctenotus dux*	Isoptera	0.57	2	2.6	2.3	10.22	8.23
*Ctenotus grandis*	Vertebrates	0.72	5	3.0	3.3	—	9.4
*Ctenotus inornatus*	Isoptera	0.55	2	2.6	2.1	10.02	10.09
*Ctenotus pantherinus*	Isoptera	0.10	2	3.2	2.1	9.51	7.02
*Ctenotus piankai*	Pentatomidae	0.79	2	2.3	2.3	—	8.78
*Ctenotus quattuordecimlineatus*	Isoptera	0.75	2	2.5	2.2	13.09	9.42

Comparisons with mean δ^15^N show mismatches in species order based on predicted trophic rank and actual trophic position based on isotopic analysis.

There was no phylogenetic signal for δ^13^C or δ^15^N values from the pooled dataset. For δ^13^C, the maximum likelihood estimate of Pagel's λ was approximately zero (λ = 5.75 x 10^−5^); using a likelihood-ratio test, this value is not significantly different from a null model with no phylogenetic signal (*P* = 1 for λ_free_ versus λ = 0). The maximum likelihood estimate of Pagel's λ for δ^15^N was also approximately zero (λ = 5.74 x 10^−5^), and not significantly different from the null model (*P* = 1 for λ_free_ versus λ = 0). Additionally, there was no phylogenetic signal for isotopic values when separated by community (Lorna Glen: λ = 7.235 x 10^−5^ for δ^13^C, λ = 7.235 x 10^−5^ for δ^15^N, *P* = 1 for λ_free_ versus λ = 0; Yamarna: λ = 5.92 x 10^−5^ for δ^13^C, λ = 5.92 x 10^−5^ for δ^15^N, *P* = 1 for λ_free_ versus λ = 0). We note that tests of phylogenetic signal may have relatively low power for small phylogenies [36]. However, we consider it unlikely that low statistical power is driving these relationships given that maximum likelihood estimates of λ converged on zero (e.g., λ < 0.001) for all analyses.

**Fig 5:**
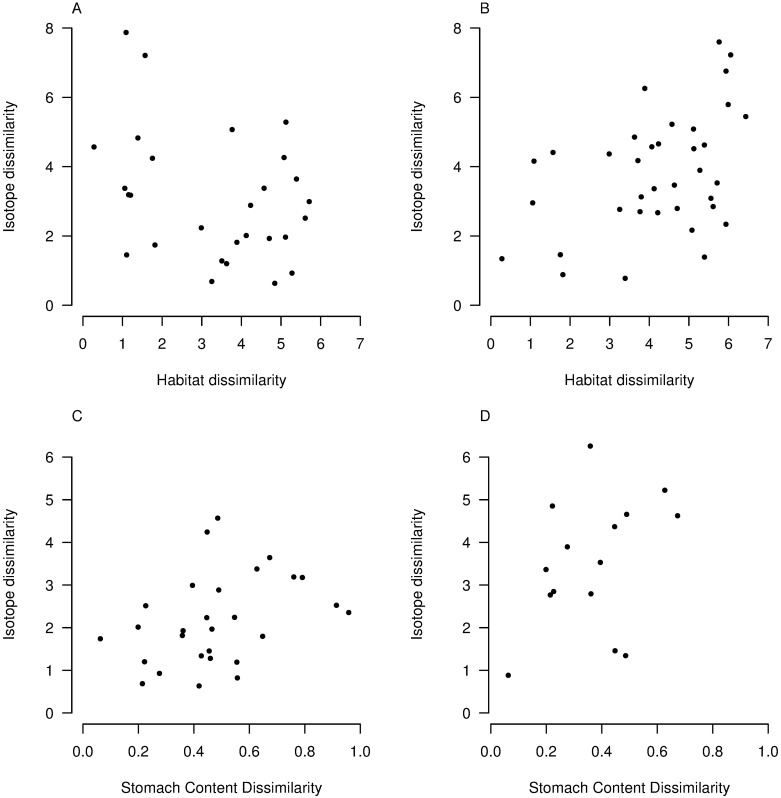
Pairwise distances in isotopic and ecological space for species from Lorna Glen and Yamarna regional communities. Euclidean distances in isotope vs. habitat space are uncorrelated for (A) Yamarna species (*r* = -0.35, *p* = 0.98) and are positively correlated for (B) Lorna Glen species (*r* = 0.43, *p* = 0.01). Euclidean distances in isotope vs. diet space are positively correlated for (C) Yamarna species (*r* = 0.37, *p* = 0.05) and (D) Lorna Glen species (r = 0.37, *p* = 0.05).

We found a significant positive relationship between pairwise distances in isotopic and habitat space for species of Lorna Glen (*r* = 0.43, *p* = 0.01) (Fig 5B). We distinguished the contributions of carbon and nitrogen by performing separate Mantel tests for δ^15^N and δ^13^C. We found no relationship between species’ distance for δ^13^C values and species’ distance in habitat space, while distances for δ^15^N and habitat values result in an *r* = 0.44 with *p* = 0.01. The correlation between isotopic composition and habitat use is strengthened after removing the non-*Ctenotus* taxa, which are ecologically distinct from the *Ctenotus* species that comprise the majority of sphenomorphines in the community (*r* = 0.63, *p* = 0.01; N = 7). The significant positive relationship between isotopic signature and habitat use for all species persists after accounting for autocorrelation based on phylogenetic relatedness using a phylogenetically permuted Mantel test (*r* = 0.44, *p* = 0.048); however, the relationship for only *Ctenotus* species becomes nonsignificant (*r* = 0.63, *p* = 0.31; N = 9). There is no relationship between distances in isotopic and habitat space for Yamarna species (*r* = -0.35, *p* = 0.98) (Fig 5A), and these results hold when comparing only *Ctenotus* species and when conducting a phylogenetically permuted Mantel test (*r* = -0.40, *p* = 0.93; *r* = 0.11, *p* = 0.87).

We found a significant and positive relationship between species’ distances in isotopic and dietary space for both Lorna Glen (r = 0.37, *p* = 0.05) (Fig 5D) and Yamarna (r = 0.37, *p* = 0.05) (Fig 5C) communities, but these results were not significant when the null distribution was constructed using phylogenetically-informed permutations (*r* = 0.67, *p* = 0.14; *r* = 0.61, *p* = 0.13). However, we note that phylogenetically-permuted Mantel tests have been shown to exhibit low power [39]. Computing distance with termites as the sole prey category (proportions in diet ranging from 10–75% for species in a dataset from ERP, Table 3) revealed a significant correlation with distance in isotopic space for Yamarna species (*r* = 0.73, *p* = 0.02), b b but not Lorna Glen. Additionally, species’ ordering along the δ^15^N axis did not match predictions based on calculations of weighted trophic rank for either community (Lorna Glen: *r* = -0.20, *p* = 0.70; Yamarna: *r* = 0.35, *p* = 0.39), although trophic spread was relatively consistent between estimates based on stomach content and isotopic signature for Yamarna (Table 3, Fig 4).

We found no significant relationship between species pairwise distances in diet and habitat space (Mantel test: *r* = -0.26, *p* = 0.86; phylogenetically permuted Mantel test: *r* = 0.19, *p* = 0.6).

## Discussion

Isotopic signatures demonstrate that sphenomorphine skinks of a diverse community from the western Australian arid zone are differentiated in a stable isotope space defined by δ^13^C and δ^15^N, implying that isotopic composition is tracking an underlying set of species-specific ecological attributes. We find that differences between species are correlated with habitat and unrelated to phylogeny at the scale of this study. Additionally, while empirical data on gut content analysis are shown to function as a moderate predictor of isotopic content, stable isotope analysis may suggest alternative dietary habits in some species in addition to variation among sites. Our study shows that stable isotope signatures could be a useful but imperfect surrogate for identifying ecological differentiation among lizard species in the Australian deserts. However, we did not find evidence that trophic position as inferred from stomach content analysis is correlated with isotopic estimates of trophic position based on δ^15^N signatures.

### Stable isotopes and stomach content analysis

Previous studies using stomach content analyses have found separation in dietary habits among *Ctenotus* species [20,26]. Our results from stable isotope analysis confirm that species appear differentiated in diet space, and suggest that this differentiation persists through time, given the timescales over which isotopic assimilation and turnover occur in reptile tissue [15–16]. Stable isotope signatures of the species in this study are comparable to values reported for reptiles in existing analyses [15–16,43]. While δ^15^N and δ^13^C values for prey items in the GVD are not available for comparison to signatures of their consumers, previous studies on detrital and grazing food webs in desert and other terrestrial ecosystems show that isotope signatures provide reliable measures of trophic links in these communities, and are subsequently detectable in higher order consumers [43–45]. These studies also demonstrate fine scale variation among prey items of the same trophic order that represent differences in grazing among C_3_ and C_4_ plant types, and that this variation is reflected by the spread of intraguild isotopic signatures in predators, similar to patterns of differentiation along the δ^13^C axis detected by the present study (Fig 1).

Despite a significant correlation between similarity in isotopic signature and similarity in dietary habits for both communities, much of the variation remains unexplained (Fig 5C and 5D). Isotopic composition better predicts dietary habits for the Yamarna community, from which data for stomach content analyses were collected. This is congruent with our results suggesting that intraspecific dietary habits differ between sites (Fig 3). Isotopic signatures for Yamarna species are correlated with the proportion of termites in a species’ diet, and are better matched to the trophic spread predicted by stomach content analysis than Lorna Glen signatures (Fig 4). However, for some species, the weighted trophic rank was a poor predictor for isotopic composition in both communities. For example, stomach content analyses indicate that *C*. *quattuordecimlineatus* feeds mostly on first and second order consumers (weighted trophic rank between 2 and 3), while the high δ^15^N values of this species from both Yamarna and Lorna Glen suggest a diet of tertiary or higher consumers, 3–4 trophic transfers (at 2.54‰ [41]) from the base of the food chain. Likewise, mismatches between the ordering of species along the δ^15^N axis and their weighted trophic rank relative to other species occurs in both communities; for example, trophic positioning based on stomach content analysis implies that *C*. *grandis* feeds almost exclusively at one full trophic level above *C*. *inornatus*, but the mean δ^15^N signatures of these species in the Yamarna community imply that they feed at roughly the same trophic level (and the mean δ^15^N signature of *C*. *grandis* is in fact lower than that of *C*. *inornatus*) (Table 3, Fig 4B). However, while the ordering of species along the δ^15^N axis is rearranged compared to what might be predicted from gut content analyses, most species pairs differ by less than what is expected for a single trophic transfer (2.54‰, [41]). Therefore, omnivorous species could feed more preferentially on higher or lower trophic order prey items than expected from gut content analyses, and these differences are reflected by the intraguild variation of sphenomorphine species in isotopic space.

Discrepancies between isotopic composition and stomach contents may be considered a reflection of biases in gut content analysis, in the relationship between isotopic composition and diet, or both. Stomach content analysis typically provides a “snapshot” view of an organism’s diet for a particular point in time and space, and may include biases of digestion rate and identification accuracy [14,46–48]. Likewise, isotopic data may not accurately reflect dietary patterns due to sampling error (e.g., small sample sizes), fractionation differences among species or prey items, or external sources of variation [49–50]. Furthermore, stomach content data came from sites at Yamarna that excluded certain habitats common to both regions (e.g. *Acacia*-dominanted shrubland). Because signatures from these habitat types may be reflected in some of the isotopic variation, this incongruence may explain the lack of strong correlation between isotopic data and stomach contents. Additionally, temporal incongruences among the datasets may reflect variation introduced by environmental change. Wildfires in the arid Australian interior can cause major turnover in plant communities, and lizard diets from the Great Victoria Desert have been shown to fluctuate during fire succession cycles [20,51]; discrepancies between isotopic data and stomach content data collected for this study during different years may well be due to fine-scale ecological variation associated with successional history.

### Stable isotopes and habitat use

For the species of Lorna Glen, variation in δ^15^N isotopic signature is correlated with divergence in habitat use, such that species closer in habitat space are also more similar in nitrogen isotopic composition. Because we lack isotopic data for prey items for the communities considered here, the present study cannot say definitively whether increased similarity in isotopic composition is directly attributable to increased similarity in diet. Correspondingly, all species could be differentiated in diet and isotopic similarity is potentially attributable to habitat-specific differences in baseline δ^15^N signatures, due to differential nitrogen-fixing abilities of vegetation. However, this explanation cannot account for the lack of association between δ^13^C and habitat. Moreover, discrepancies between habitat variables and isotopic signature may reflect successional changes between the time of collection for habitat and isotope data, as discussed above.

Despite the absence of a significant relationship between δ^13^C composition and habitat use, changes in isotopic composition with individual habitat variables reveal predictable patterns of variation. Sites in the study region are characterized by a mosaic of habitats with dominant vegetation types that differ in their use of C_3_ and C_4_ photosynthetic pathways; among these are *Acacia* groves (C_3_), spinifex sandplains (C_4_), and chenopod shrublands (C_4_). Because plants that utilize a C_4_ photosynthetic pathway discriminate less strongly against the heavy stable isotope of carbon (^13^C) than C_3_ plants, C_4_ plants tend to exhibit δ^13^C signatures between -17 to -9‰, while C_3_ plants exhibit δ^13^C signatures between -32 to -22‰ [42]. Since carbon is not significantly enriched through trophic transfers [4,52], prey items found in different habitats will reflect the baseline signatures of primary producers [4]. Because most species’ means for δ^13^C are intermediate between the ranges expected for C_3_ and C_4_ plants, skinks of these communities seem to be feeding on prey items that utilize both types of vegetation, or on predators that utilize two types of prey with differing C_3_/C_4_ phytophagy preferences. Species strongly associated with spinifex grasses (*C*. *pantherinus*, *C*. *calurus*, *C*. *inornatus*, *C*. *quattuordecimlineatus*, *C*. *grandis* [1]), which utilize a C_4_ photosynthetic pathway, exhibit higher δ^13^C signatures than species that are more strongly associated with *Acacia* woodlands (*C*. *schomburgkii* and *C*. *leonhardii* [1]), in which the trees utilize a C_3_ photosynthetic pathway (Welch’s two sample t-test, *p* = 0.03). Additionally, *Acacia* plants are nitrogen-fixing, resulting in soil and litter that is enriched in δ^15^N [42]; and the two species associated with this habitat demonstrate some of the highest δ^15^N signatures (Table 2).

### Variation between sampling sites

In addition to revealing how dietary differentiation persists through time, results from stable isotope analysis show that species separation is also consistent among sites; however, several species from Yamarna demonstrate a significantly lower signal for δ^15^N values than the same species collected at Lorna Glen. A greater diversity or abundance of predatory arthropods in the Lorna Glen community might contribute to the higher δ^15^N signatures exhibited by species from this site. Such hypotheses are speculative, although nearby regions have been observed by the authors to show some variation in macroinvertebrate communities. Alternatively, potential differences in successional history between the two regions could have resulted in different plant communities [20], which could in turn allow for variation in populations of lower trophic order prey items like termites (in addition to promoting variability in δ^13^C signatures among sites). While plant communities of the two study regions are broadly comparable, we lack quantitative data necessary to assess variation. Thus, the present study is unable to distinguish whether inconsistencies in signatures between sites reflect variation in baseline resource values of δ^15^N and δ^13^C, or a change in food web structure [53], and isotopic signatures of consumer prey species from each site are recommended for accurate comparisons of trophic position [53].

Recent advancements in stable isotope analysis have enabled the comparison of ecological groups using multivariate ellipse-based metrics in a Bayesian framework, eliminating uncertainty derived from small sample sizes and allowing for robust statistical comparison among communities by accounting for natural variability within the system [54]. These descriptive metrics, expanded from a previous study [5], can be used as measurements of niche structure and incorporated with other axes of ecological information to obtain more comprehensive estimates of niche hypervolume. However, the lack of strong structure exhibited by isotopic data along ecological axes of diet and habitat in the present study, in addition to the lack of coordination between datasets, could make inference of niche width from species’ arrangements in isotopic space somewhat unreliable. Obtaining isotopic signatures for the prey items available to Sphenomorphine skinks in the GVD would also be beneficial, and would allow researchers to exploit isotope mixing models that would provide more precise estimates of the relative contribution of each prey category to an individual’s diet [55].

## Conclusion

This study demonstrates that stable isotope analysis is useful in revealing patterns of species separation in the hyperdiverse lizard communities of the western Australian arid zone. Despite intraspecific variation in resource use and interspecific similarities in prey preference, our results show that stable isotopes can reveal patterns of trophic differentiation within and between sites when combined with stomach content analysis. These methods are an attractive complement to high-resolution analyses of individual stomach contents (e.g., [20,24], etc.) as they potentially provide a temporally integrated index of trophic ecology in reptile communities. However, much variation remains unexplained in our study, highlighting the need for a more comprehensive isotopic analysis of both prey and consumer species. The trophic ecology of lizard communities of the Great Victoria Desert and adjacent regions is perhaps as well studied as any squamate reptile assemblage [21–24], yet the incongruences noted here suggest that much remains to be learned about this and other systems.

## Supporting information

S1 File Habitat data from Rabosky et al. (2011) *American Naturalist*. This supplementary file is a previous publication containing habitat data used in the present study.
(PDF)

S2 File Stable isotope and stomach content data used in the present study. (ZIP)
