# Supplementary material for: Stable isotope ecology of a hyper-diverse community of scincid lizards from arid Australia
Source: PLoS One. 2017 Feb 28;12(2):e0172879. doi: 10.1371/journal.pone.0172879 (PMC5330509; doi:10.1371/journal.pone.0172879)
Supplement: S1 File — This supplementary file is a previous publication containing habitat data used in the present study. (PDF) [file pone.0172879.s001.pdf]

Species Interactions Mediate Phylogenetic Community Structure in a Hyperdiverse Lizard Assemblage from Arid Australia.
Author(s): Daniel L. Rabosky, Mark A. Cowan, Amanda L. Talaba, Irby J. Lovette
Reviewed work(s):
Source: *The American Naturalist*, Vol. 178, No. 5 (November 2011), pp. 579-595
Published by: The University of Chicago Press for The American Society of Naturalists
Stable URL: http://www.jstor.org/stable/10.1086/662162
Accessed: 10/11/2011 17:34

Your use of the JSTOR archive indicates your acceptance of the Terms & Conditions of Use, available at
http://www.jstor.org/page/info/about/policies/terms.jsp

JSTOR is a not-for-profit service that helps scholars, researchers, and students discover, use, and build upon a wide range of content in a trusted digital archive. We use information technology and tools to increase productivity and facilitate new forms of scholarship. For more information about JSTOR, please contact support@jstor.org.

The University of Chicago Press and *The American Society of Naturalists* are collaborating with JSTOR to digitize, preserve and extend access to *The American Naturalist*.

http://www.jstor.org

VOL. 178, NO. 5 THE AMERICAN NATURALIST NOVEMBER 2011

Species Interactions Mediate Phylogenetic Community Structure in a Hyperdiverse Lizard Assemblage from Arid Australia

Daniel L. Rabosky,[1,2,3,*] Mark A. Cowan,[4] Amanda L. Talaba,[2] and Irby J. Lovette[2,3]

1. Department of Integrative Biology and Museum of Vertebrate Zoology, University of California, Berkeley, California 94720; 2. Fuller Evolutionary Biology Program, Cornell Laboratory of Ornithology, Ithaca, New York 14850; 3. Department of Ecology and Evolutionary Biology, Cornell University, Ithaca, New York 14853; 4. Science Division, Department of Environment and Conservation, P.O. Box 51, Wanneroo, Western Australia 6946, Australia

Submitted March 7, 2011; Accepted June 10, 2011; Electronically published September 29, 2011

Online enhancements: appendix, zip files.

ABSTRACT: Evolutionary history can exert a profound influence on ecological communities, but few generalities have emerged concerning the relationships among phylogeny, community membership, and niche evolution. We compared phylogenetic community structure and niche evolution in three lizard clades (*Ctenotus* skinks, agamids, and diplodactyline geckos) from arid Australia. We surveyed lizard communities at 32 sites in the northwestern Great Victoria Desert and generated complete species-level molecular phylogenies for regional representatives of the three clades. We document a striking pattern of phylogenetic evenness within local communities for all groups: pairwise correlations in species abundance across sites are negatively related to phylogenetic similarity. By modeling site suitability on the basis of species' habitat preferences, we demonstrate that phylogenetic evenness generally persists even after controlling for habitat filtering among species. This phylogenetic evenness is coupled with evolutionary lability of habitat-associated traits, to the extent that closely related species are more divergent in habitat use than distantly related species. In contrast, lizard diets are phylogenetically conserved, and pairwise dietary overlap between species is negatively related to phylogenetic distance in two of the three clades. Our results suggest that contemporary and historical species interactions have led to similar patterns of community structure across multiple clades in one of the world's most diverse lizard communities.

Keywords: community structure, phylogeny, diversification, competition, adaptive radiation, niche conservatism, species tree.

Introduction

As recognized by Darwin (1859), closely related species may show ecological similarity as a result of shared evolutionary history. This ecological similarity may have a range of consequences for patterns of community structure, depending on the relative importance of interspecific competition, environmental filtering, differences in competitive abilities among species, and other factors (Webb 2000; Webb et al. 2002; Cavender-Bares et al. 2004; Kraft et al. 2007; Mayfield and Levine 2010). However, we still lack a basic understanding of empirical patterns characterizing communities of closely related species that occur together in syntopy (Vamosi et al. 2009). Yet it is these fine phylogenetic and spatial scales over which some of the most interesting dynamics of community assembly take place: the spatial scale is sufficiently fine-grained to allow the possibility of interactions between individuals, and the phylogenetic scale is such that species ecological traits may still retain the imprint of evolutionary history (Vamosi et al. 2009).

In this article, we address the role of current and historical ecological processes in structuring lizard communities in Australia's Great Victoria Desert (hereafter GVD). We use long-term ecological monitoring data in concert with species-level molecular phylogenies to assess whether the assembly of local communities from regional species pools is dominated by stochastic processes (e.g., community drift) or by contemporary and/or historical interspecific competition. This system is ideal for testing models of community assembly for a number of reasons. First, lizard diversity in the GVD is extremely high at both local and regional scales (Pianka 1969*b*, 1986; Morton and James 1988; James and Shine 2000). Diversity may be higher within particular habitat types (particularly spinifex sandplains) than in any other habitat on Earth, with more than 70 lizard species known to co-occur at single localities. This level of diversity is nearly an order of magnitude greater than that known for physiographically comparable sites in North America (Pianka 1986). Second, this diversity is drawn from replicate evolutionary radiations, such

* Corresponding author; e-mail: drabosky@berkeley.edu.

Am. Nat. 2011. Vol. 178, pp. 579–595. © 2011 by The University of Chicago. 0003-0147/2011/17805-52871$15.00. All rights reserved.
DOI: 10.1086/662162

that a number of highly divergent clades achieve comparable levels of regional and local species richness. The replicate nature of these radiations, in concert with high regional and local diversity, enables us to test whether common mechanisms underlie community structure in divergent groups of organisms that inhabit a similar ecological and biogeographic setting.

We focus on three clades of insectivorous lizards that dominate lizard communities in the GVD: skinks in the genus *Ctenotus*, diplodactyline geckos, and agamids. The three clades show high diversity at local, regional, and continental scales (Pianka 1969*a*, 1969*b*, 1986; Rabosky et al. 2007*a*, 2007*b*), and species can be found in virtually all habitats that occur in the GVD (fig. 1). Habitat filtering clearly occurs among members of these groups in the GVD: most species show high levels of habitat specificity as well as habitat-associated morphological specialization (Pianka 1969*a*, 1972; Read 1995; Melville et al. 2001, 2006; Daly et al. 2008). Although these three clades share a few general similarities in gross morphology as well as high regional species diversity, they diverged from one another more than 150 million years ago (Vidal and Hedges 2009) and thus constitute replicate examples of the interplay between evolutionary history and species interactions. The three clades are separated by differences in both diet and foraging mode (Vitt et al. 2003; Vitt and Pianka 2005). Agamids tend to be sit-and-wait predators that rely principally on visual prey detection, whereas geckos and skinks are active foragers that utilize olfactory (geckos) or vomeronasal (skinks) chemical prey detection. Moreover, agamid diets are often dominated by ants and other hymenopterans, but skinks and geckos frequently avoid these noxious prey items (Pianka 1986; Vitt et al. 2003).

To investigate phylogenetic community structure and niche evolution in these three clades, we conducted multiyear surveys of lizard communities at Lorna Glen (26°15′S, 121°20′E; figs. B1, B2, available online), a conservation reserve located in the northwestern GVD at the junction of the Murchison and Gascoyne bioregions (Environment Australia 2000). The Lorna Glen community includes at least 70 species of squamate reptiles, including 10 *Ctenotus* skinks, 11 agamids, and 11 diplodactyline geckos (fig. B5, available online). We estimated species abundances on plots ("sites") that were selected to encompass the range of habitats encountered in the Lorna Glen region. We first tested whether closely related species pairs were characterized by contrasting patterns of abundance among sites. Such negative abundance-relatedness correlations (a form of phylogenetic evenness) are expected if (1) competitive interactions between closely related species lead to local density trade-offs or (2) historical interspecific competition has resulted in divergent habitat preferences among closely related species. However, negative relationships are not expected to arise under models

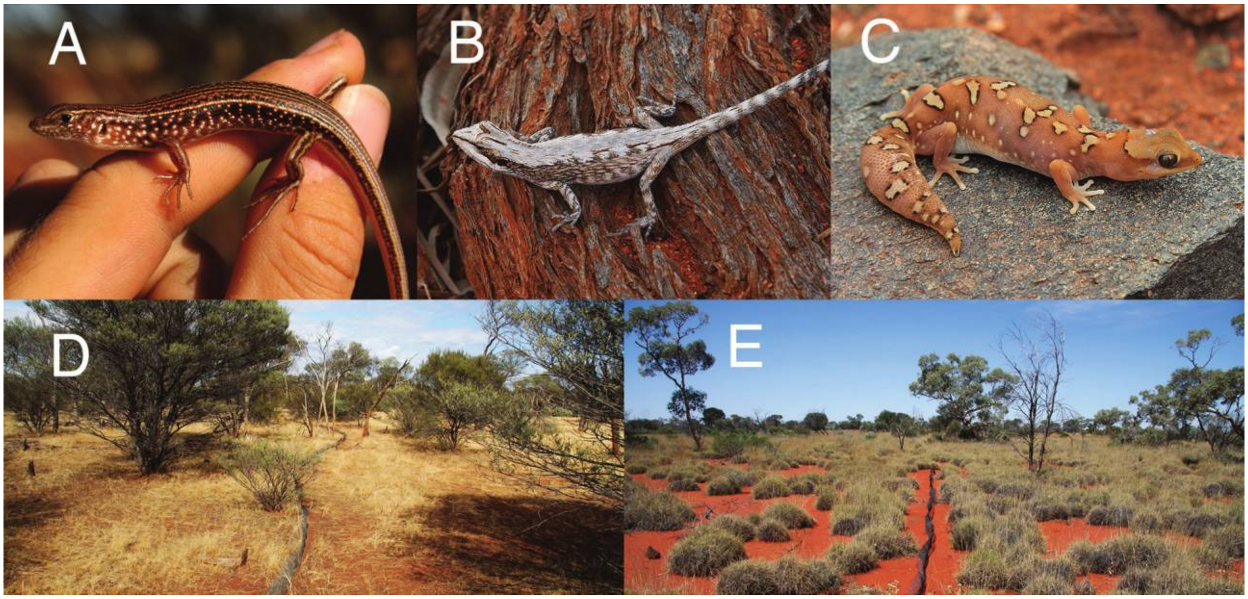

Figure 1: Representative lizards and habitats from the Lorna Glen study region. *A*, *Ctenotus uber*, a skink that occurs in chenopod and *Acacia* shrublands; *B*, *Caimanops amphibuloroides*, an arboreal agamid that occurs in *Acacia* woodlands; *C*, *Diplodactylus pulcher*, a gecko that occurs in rocky habitats; *D*, *Acacia* woodland; *E*, spinifex-dominated sandplain habitat. Drift fencing from pitfall traplines can be seen in center of photos in *D* and *E*. Photographs by D. L. Rabosky.

of community drift (Hubbell 2001), which predict patterns of phylogenetic clustering or randomness within local communities (Kraft et al. 2007).

These explanations are not mutually exclusive, because species abundances within any particular habitat might be governed by community drift, even if broad-scale patterns of habitat use have been shaped by trait divergence in response to interspecific competition. Likewise, interspecific competition may still govern the dynamics of community assembly among species with shared habitat preferences (e.g., Helmus et al. 2007), regardless of the processes that generated the overall distribution of habitat preference. To distinguish between these possibilities, we tested observed community structure against an environmentally constrained null model that explicitly accounted for habitat filtering. This enabled us to ask whether phylogenetic clustering or evenness was observed within local communities after accounting for evolutionary divergence in species' habitat preferences. Finally, we modeled the evolution of ecological niche parameters to understand how divergence and conservatism in habitat and diet relate to observed patterns of species co-occurrence and abundance.

Methods

Community Structure Data

We have been conducting long-term monitoring of lizard populations at Lorna Glen in central Western Australia; the study region consists of approximately 7,000 km^2 of sandplain, dune, *Acacia* savannah, saltbush, and other habitats. We conducted two separate surveys between 2001 and 2007. For the first survey, hereafter LG24, we established linear arrays of 12 pitfall traps on 24 0.25-ha plots throughout the reserve. The 24 focal sites were selected to encompass the full range of habitats within the Lorna Glen region (fig. 1). For the second survey, hereafter LG8, we focused primarily on the spinifex (*Triodia*, "porcupine grass"; fig. 1; also fig. B3, available online) sandplain communities that harbor the most diverse lizard assemblages in the region. Each of the eight sites in the LG8 survey consisted of 10 arrays of four pitfall traps distributed across 3 ha. In contrast to LG24, in LG8 sites were situated exclusively in sand communities. Most traps in LG8 were situated in spinifex sandplain habitats of different successional stages (fig. B3), although a small percentage of traps were placed on dunes. In both LG8 and LG24, sites were separated by approximately 3–10 km.

The LG8 and LG24 sites were surveyed multiple times per year for continuous multiday intervals between 2001 and 2007, for totals of 39,744 and 51,200 trap nights at LG24 and LG8, respectively. During a given survey period, sites in either LG24 or LG8 were sampled simultaneously, such that all sites within each survey received precisely the same sampling effort. Most animals were released after capture, but multiple representatives of each species were deposited in collections at the Western Australian Museum and the Cornell Museum of Vertebrates to verify and validate field identifications. Across both LG8 and LG24, we recorded captures of 2,102 *Ctenotus* skinks, 587 agamids, and 2,660 diplodactyline geckos. Although we did not conduct a comprehensive mark-recapture study, we believe that our total number of recorded captures provides a good approximation of relative species abundance for several reasons. Limited mark-recapture surveys suggest low overall recapture rates (M. A. Cowan and D. L. Rabosky, personal observation). Furthermore, we removed all *Ctenotus* skinks captured in one 7-day survey period as part of another study; this removal had no effect on subsequent capture rates (fig. B4, available online). Species from the three focal radiations (*Ctenotus* skinks, agamids, and geckos) accounted for the majority of squamate reptile captures at Lorna Glen during the survey period and included eight of the 10 most frequently encountered species (fig. B5).

In the analyses presented here, we focus exclusively on community structure and ecological niche evolution within these clades. We ignore interactions that may be occurring between these clades as well as those that potentially occur between our focal species and members of other lineages of squamates (including varanids, snakes, pygopodids, *Lerista* skinks, and *Egernia*-group skinks; see fig. B5), but it is nonetheless possible that such interactions have contributed to patterns of resource use and abundance in the clades we study. Our analyses of trait evolution and community structure within the focal clades are robust to the omission of other taxa, provided that we are explicit about the phylogenetic scale to which our conclusions apply (e.g., our analyses of ecological niche evolution in *Ctenotus* skinks will still be valid with respect to *Ctenotus*, even if the patterns we report for *Ctenotus* break down at more inclusive scales).

Our analyses of phylogenetic community structure (see below) assume that (1) all species that occur within the regional pool have been detected and (2) dispersal limitation via biogeographic barriers does not preclude species from reaching some sites within the study region. All species from the three radiations have broad geographic distributions that extend beyond the boundaries of Lorna Glen in all directions, and there are no parapatric contacts (or allopatry) between any species within the study region. We believe that all sites in our survey are accessible to all species in the regional pool via dispersal over timescales of several generations; results of this survey and more detailed surveys targeting particular habitats (D. L. Ra-

bosky, personal observation) suggest that all species occur within several kilometers of every site on Lorna Glen. Analysis of species accumulation curves suggests that all species were detected quickly after the initiation of sampling (fig. B6, available online). Moreover, analysis of museum records and geographic distributions for other arid-zone reptiles suggest that all species that should have been present were in fact detected during the survey period.

Phylogenies

Using Bayesian methods, we estimated complete species-level phylogenies for all members of the three focal radiations present in the Lorna Glen regional community. To reconstruct a phylogeny for *Ctenotus* skinks, we sequenced introns from four nuclear loci (ornithine decarboxylase [ODC; 600 bp], ATP synthetase beta-subunit intron [ATPSB; 550 bp], glyceraldehyde 3-phosphate dehydrogenase intron 11 [GAPDH; 650 bp], and rhodopsin 2 intron 3 [RHO; 1,300 bp]) and two mitochondrial genes (NADH subunit 4 [ND4; 650 bp] and cytochrome B [cytB; 1,143 bp]), comprising a total of 5,408 aligned base pairs. The *Ctenotus* individuals sequenced were collected at the Lorna Glen community. For the agamids and geckos, we downloaded all phylogenetically informative sequence clusters for taxa present in the Lorna Glen community from GenBank, using the PhyLoTA browser (Sanderson et al. 2008). Available sequence data for both groups consisted of the mitochondrial DNA (mtDNA: NADH subunit 1 [ND1], NADH subunit 2 [ND2], and several intervening transfer RNAs [tRNAs]). We evaluated a range of partitioning strategies for the data (Brandley et al. 2005), and model selection for each partition was performed with MODELGENERATOR (Keane et al. 2006). Additional details on molecular methods and phylogenetic analysis are given in Rabosky et al. (2009) and appendix A, available online.

Because we had multilocus sequence data for skinks, we inferred species trees by using a Bayesian hierarchical species-tree model as implemented in BEST v1.0 (Liu 2008). This approach estimates the overall phylogenetic tree (e.g., the "species tree") that can best explain the distribution of gene trees observed for independent loci, by assuming that loci are correlated by their shared species history (Edwards et al. 2007; Liu and Pearl 2007). The method uses a coalescent framework to approximate the posterior distribution of species trees conditional on the gene trees, the coalescent model, and the sequence-evolution models used to infer the gene trees. The species-tree framework accounts for the fact that incomplete lineage sorting can lead to discord between individual gene trees and the species tree and can provide estimates of species trees that are more robust than those from widely used concatenation approaches (Edwards et al. 2007; Leaché and Rannala 2010). Species-tree modeling in BEST requires specification of prior distributions on relative mutation rates across all loci (μ) as well as the effective-population-size parameter (θ). Following Leaché (2009), we conducted a series of preliminary Markov chain Monte Carlo (MCMC) runs in BEST to identify appropriate prior distributions for θ; final analyses used an inverse gamma distribution prior on θ with a mean of 0.05 ($\alpha = 3$, $\beta = 0.1$) and a uniform distribution prior on μ (range 0.05–5). For our final analyses, we conducted three MCMC runs of 250 million generations each, sampling species and gene trees every 20,000 generations.

We assessed convergence of MCMC runs in AWTY (Nylander et al. 2008) by assessing the stability of posterior probabilities on individual nodes as well as the correspondence in posterior probabilities among independent runs. For agamids and geckos, phylogenies were estimated with relaxed-clock methods implemented in BEAST (Drummond et al. 2006; Drummond and Rambaut 2007), as we did not have multilocus data available for those radiations. We did not incorporate fossil calibrations into our analyses; trees are therefore calibrated on a relative, rather than an absolute, timescale. The timing of the oldest split within each of the focal clades is surrounded by considerable uncertainty, but it probably occurred 10–15 million years ago (Ma) for *Ctenotus* (Rabosky et al. 2007*b*; Skinner et al. 2011), roughly 20–25 Ma for the agamids (Hugall et al. 2008), and perhaps 30–70 Ma for the diplodactylines (Oliver and Sanders 2009). We do not believe that the difference in the ages of these radiations is problematic for our analyses, as our primary focus is to explore patterns within clades. However, in "Discussion," we consider age variation as one factor that might explain why a few patterns vary between these clades. Multiple BEAST runs were conducted for 50 million generations, with trees sampled every 5,000 generations. Convergence was assessed in AWTY as described above and by evaluating the effective sample sizes of molecular evolutionary parameters with TRACER (Drummond and Rambaut 2007). Sample and GenBank accession numbers are given in tables A1–A3, available online.

Habitat Data

Fourteen habitat variables were measured/estimated for each of the 928 pitfall traps in LG24 and LG8. Within a 3-m radius of each trap, we visually estimated the percentage of ground covered by (1) hummock grasses (spinifex; *Triodia* sp.), (2) tussock grasses, (3) low shrubs, especially chenopods, (4) tree canopy cover, and (5) total sheltering vegetation. We estimated the percentage of exposed ground that was (6) sand or earth, (7) gravel, and

(8) rocks with a diameter of >2 cm. We further measured (9) soil compaction, using a soil penetrometer, and (10) soil shear strength, using a torsional-vane shear tester. Finally, we measured (11) the volume of woody debris on the ground within 3 m of each trap, (12) the distance from each trap to the nearest sheltering vegetation, (13) the diameter of the largest sheltering vegetation within 3 m of each trap, and (14) the distance to the nearest tree with a diameter at breast height of >5 cm. Precipitation, temperature, and humidity do not vary markedly across Lorna Glen, and we did not attempt to quantify climatic differences associated with each site.

Community Structure Analyses

To explore the phylogenetic structure of desert lizard communities, we tested whether phylogenetic distance between species predicted their pairwise covariation in abundance across all sites in the LG24 and LG8 data sets. We first computed the Spearman correlation in abundance between all species pairs within each clade (Ricklefs 2011), generating a matrix of pairwise rank correlations in abundance between individual species pairs. We then tested whether this matrix of abundance correlations was significantly associated with the pairwise-distance matrix derived from species' pairwise phylogenetic distances. Although many powerful tests have been developed to describe local community structure (Webb 2000; Cadotte et al. 2009; Ives and Helmus 2010), we chose the Spearman correlation because it is a simple metric that enabled us to summarize patterns across multiple sites in our full data set while using relative-abundance information. Such summary statistics may be especially appropriate for our data, because sampling effort was constant across sites: all sites within each data set were sampled simultaneously. Moreover, the use of a rank correlation is well suited to our estimates of relative abundance, where the observed "abundance" at particular sites is potentially inflated by recaptured individuals (but see fig. B4). If recapture rates are identical for all species within major clades, then our measures of pairwise species associations will be identical to those we would observe if we had estimates of absolute abundance.

A positive matrix correlation between pairwise phylogenetic distances and pairwise abundance correlations thus indicates phylogenetic evenness, because it implies that closely related species show contrasting patterns of abundance across sites relative to distantly related species. This matrix correlation is expected to be approximately 0 in the absence of phylogenetic clustering or evenness. To demonstrate this, we randomly generated 5,000 community data sets from the LG24 data by reshuffling species abundances among sites with the independent-swap algorithm (Gotelli 2000), preserving the number of site occurrences per species, the number of species per site, and the total abundance of each species across all sites. We found that the distributions of matrix correlations under this null model were centered on 0 for each clade (*Ctenotus*, $r = 0.005$; agamids, $r = 0.003$; geckos, $r = -0.002$).

Although the absolute value of individual pairwise correlations in abundance may be constrained in multispecies matrices (Brown et al. 2004), note that we are not interested in detecting individual species pairs with significant positive or negative relationships. We assessed significance with Mantel tests, which have recently been criticized as having low statistical power in some phylogenetic comparative analyses (Harmon and Glor 2010). However, this test does not appear to suffer from elevated Type I error rates, particularly when applied to pairwise analyses of species abundance (Ulrich and Gotelli 2010).

The Mantel test cannot distinguish between contrasting patterns of abundance resulting from divergent or shared habitat preferences; this is true for any null model of community structure that does not explicitly account for habitat filtering during the assembly of local communities from regional species pools. We thus developed an environmentally constrained model (Peres-Neto et al. 2001; Peres-Neto 2004) that enabled us to generate null communities where species abundances were assigned probabilistically to sites on the basis of habitat suitability of a given site for a particular species. Denote the total abundance of species i, summed across all sites, as α_i. If we can estimate the probability that a given site s is suitable for species i, then we can assign the α_i individuals to sites on the basis of the vector $\mathbf{p}$ of site probabilities to generate a null distribution of species abundances across sites that is explicitly conditioned on species-specific habitat preferences. We can compute the relative probability, or weight, of site s as a function of the n habitat variables, or

$$W(s) = \prod_{k=1}^{n} f_{i,k}(x_{s,k}),$$

where $x_{s,k}$ is the measured value of habitat variable k for site s and $f_{i,k}$ is the probability density function (PDF) for habitat variable k and species i.

We estimated density functions for all species by using kernel density estimation (KDE), a nonparameteric method of inferring PDFs that makes no assumptions about the underlying shape of the distribution. These methods are increasingly used in evolutionary ecology for applications ranging from home range estimation (Seaman and Powell 1996) to analyses of body size evolution (Rabosky et al. 2007*a*; Adams and Church 2008). Given that we have a set of habitat measurements associated with

each individual lizard capture, it is straightforward to envision a histogram describing the relative density of lizard captures for all possible values of a given habitat variable *k*. KDE explicitly converts this to a PDF by overlaying a formal distribution, referred to as the smoothing kernel, on each observation. The value of $f_{i,k}(x)$ is simply the summed density at any point x, taken over all individual kernels.

The critical parameters for KDE are the choice of smoothing kernel and the bandwidth of the smoothing kernel. Here, we used a Gaussian kernel and evaluated a range of possible bandwidths (e.g., the standard deviation of the smoothing kernel). If the bandwidth is too small, the resulting PDF will have too many peaks and will be dominated by the noise in the data; if the bandwidth is too large, the PDF will become uninformative, ultimately approaching a uniform distribution as the bandwidth goes to infinity. The effect of bandwidth choice on estimated PDFs for different habitat variables is shown in figure 2. We used two criteria for selecting an optimal bandwidth (fig. B7, available online); both criteria suggested that a value of 0.15 (fig. 2, middle row) best explained major features of the observed data. Because we measured habitat variables at the level of individual pitfall traps, we computed weights separately for each pitfall trap and averaged these "trap weights" within sites to generate mean weights for each site in the LG8 and LG24 data sets.

To simulate null communities under the environmentally constrained model, individuals from the total species abundance count α_i are assigned to sites with probability proportional to $W(s)$ until all captured individuals have been assigned to sites. If habitat filtering is the only process influencing the distribution of abundance among sites, then communities simulated in this fashion should be similar to the observed community. One advantage of this approach is that site suitability is a function only of species presence data, not of species absences. Thus, if a species fails to occur at a particular site s because of local competitive interactions, the site can still have a very high likelihood of occupancy if it is similar to other sites where the species occurs, and the species might occur at site s with high frequency in null communities. Note that this approach assumes that site attributes combine independently to determine site suitability for a given species, and

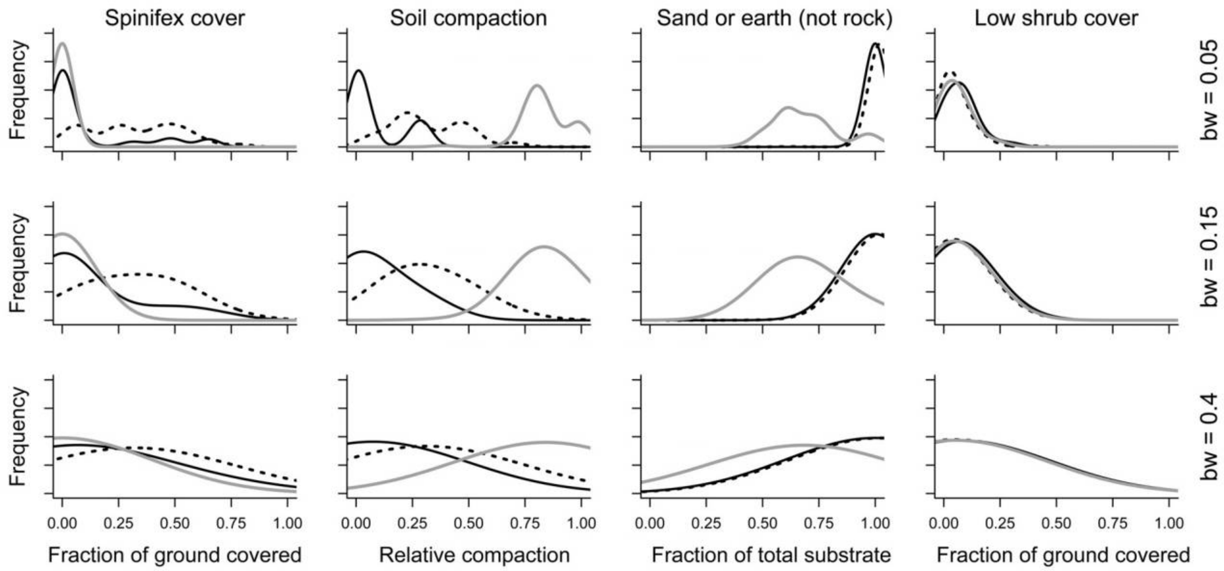

Figure 2: Estimated probability density functions (PDFs) for three species and four habitat variables, inferred via kernel density methods. Shown are PDFs for *Ctenotus quattuordecimlineatus* (skink; solid black lines), *Ctenophorus isolepis* (agamid; dashed lines), and *Diplodactylus pulcher* (gecko; gray lines). Each plot depicts PDFs for a particular habitat variable (columns) and bandwidth (bw) of smoothing kernel (rows). Excessively small bandwidths are dominated by noise in the data, and large bandwidths can be uninformative. The contrasting habitat preferences of the focal species are readily visualized in the plots above. *Ctenotus quattuordecimlineatus*, a dune specialist, prefers sites with loose sand (columns 2 and 3) and minimal spinifex cover (column 1). *Ctenophorus isolepis*, a spinifex sandplain specialist, prefers somewhat harder sand surfaces with moderate spinifex cover. *Diplodactylus pulcher* utilizes extremely stony, hard substrates and does not occur in spinifex habitats. All three species use habitats with minimal shrub cover.

it is possible that explicitly incorporating covariation in site attributes into the weight calculations could improve occurrence predictions. However, such covariation should not be problematic for our analyses. If a site is estimated to have a high weight for a particular species, any other species with similar habitat attributes should also have a high weight for that site. The null model will thus predict high co-occurrence between species with similar habitat attributes, even if (in the extreme case) it predicts their occurrence at sites that are marginal for either species. The key feature of the habitat-weighted null model is its ability to predict patterns of covariation in abundance across sites for species on the basis of their similarity in habitat use, rather than the precise sites at which any given species occurs.

For each null community simulated in this fashion, we computed the pairwise correlation in abundance across all sites for all species pairs; we then computed the matrix correlation between these correlations and pairwise phylogenetic distances for each clade. These matrix correlations represent the expected relationship between the pairwise correlation in species abundance and phylogenetic distance under a model of community assembly by habitat filtering alone. Observed coefficients were then compared to the distribution tabulated from 2,000 such simulations. For analyses of both data sets (LG24 and LG8), we excluded taxa known only from a single site, because we found that estimates of pairwise correlations were not robust for taxa with extremely low overall occupancy. The R code for estimating $f_{i,k}$ and simulating null communities is available in a zip file, available online.

Habitat Niche Evolution

We tested for conservatism and lability of niche parameters that described habitat usage for all species. We first computed the mean value of each habitat variable for each species over all pitfall traps where the species was captured. We transformed these mean values by using the transformation leading to the best approximation of normality (log, logit, or arcsine square root), as assessed with a Shapiro-Wilk test. We examined pairwise relationships of all variables after transformation to identify and exclude those showing consistent nonlinear relationships with other variables that could not be removed by transformation; three variables were removed (woody debris, distance to nearest tree, and soil shear strength). Including these three variables had virtually no effect on all analyses reported below. We then conducted a principal-components (PC) analysis on the correlation matrix of species' mean values.

We used the K statistic (Blomberg et al. 2003) to test whether phylogenetic relationships predict similarity in habitat usage for species in the three focal radiations. We computed the K statistic independently for all major PC axes and assessed significance by randomizing data across the tips of the trees (Blomberg et al. 2003). A value of $K = 1$ implies that a model of trait evolution by Brownian motion explains the data well; $K < 1$ implies that species are less similar than expected under a Brownian motion model. A value of K that is significantly less than expected if data are randomly permuted across the tips of a phylogeny indicates that closely related species are more divergent than distantly related species, as might be expected if character displacement between close relatives has occurred; conversely, this pattern can be attributed to greater similarity between distant relatives due to convergent evolution (Ackerly and Donoghue 1998; Blomberg et al. 2003; Cavender-Bares et al. 2004).

Finally, we tested whether the most closely related species pairs within each clade were more similar or less similar than expected under a simple model of trait evolution by Brownian motion. We define "most closely related species pairs" as monophyletic pairs of species within the regional species pool; note that these taxa need not correspond to true sister taxa across the entire Australian radiation. We computed the Euclidean distances between PC scores for all such species pairs, retaining the minimal set of PC axes that explained at least 95% of the variation in habitat use among the focal taxa. We then fitted a model of evolution by Brownian motion to the species' PC scores, estimating both the mean and the variance of the multivariate normal distribution describing the expected distribution of phenotypes, given the observed phylogenetic variance-covariance matrix. We simulated 2,000 character data sets under these parameters and tabulated the number of simulations where the observed difference in PC scores between closely related species exceeded the simulated value. All statistical analyses and simulations were conducted in the R programming environment, with some source code modified from the vegan (Oksanen et al. 2010), GEIGER (Harmon et al. 2008), and Picante (Kembel et al. 2010) libraries.

Dietary Niche Evolution

We tested whether diets of regionally sympatric members of the focal clades are characterized by conservatism or lability. Pianka (1969*b*, 1986) presented dietary summaries for GVD lizards derived from stomach-content analyses of thousands of individual lizards. Pianka's data include nearly all species found in the Lorna Glen community and consist of mean frequencies of 19 different classes of prey items (e.g., centipedes, spiders, termites, ants, and beetles) found in lizard stomachs. To test the relationship between phylogenetic distance and dietary divergence, we computed Pianka's (1973) metric of niche overlap between

species pairs, a distance statistic that ranges from 0 (complete dissimilarity) to 1 (perfect overlap). We then conducted Mantel tests on the matrices of dietary overlap and phylogenetic distance for the three focal clades, assessing significance with 5,000 permutations.

Results

Phylogenies and Community Structure

We recovered well-supported phylogenies for *Ctenotus* skinks, agamids, and diplodactyline geckos. Our trees were generally congruent with previous phylogenetic hypotheses for these groups (Melville et al. 2001, 2004; Rabosky et al. 2007*b*). Twenty-five of the 29 total nodes were recovered with Bayesian posterior probabilities greater than 0.95 (fig. 3). Pairwise correlations in species abundance are positively related to phylogenetic distance for both geckos and skinks (fig. 4; table 1), indicating that closely related species show contrasting patterns of abundance across all sites. This relationship is not significant for agamids, considered as a whole, in either the LG24 or the LG8 data set. However, when the analysis is restricted to the "core" amphibolurine agamid radiation (Hugall et al. 2008) by excluding the species *Moloch horridus*, the pattern within agamids is significant for the LG24 data set ($r = 0.258$). There is some justification for excluding this earliest-diverging lineage, as it probably represents an independent invasion of the arid zone relative to all other Australian agamids (Hugall et al. 2008) and shows extreme ecological and morphological specialization (Pianka and Pianka 1970). In the LG8 data set, there is presumably little power to detect effects in the agamids, given that comparatively few species of agamids occurred in this data set (table 1).

For *Ctenotus* skinks and diplodactyline geckos, the correlation between pairwise abundances and phylogenetic distance is significant under the environmentally constrained null model for both the LG24 and LG8 data sets (table 1). This indicates that habitat filtering of closely related species with divergent habitat preferences cannot

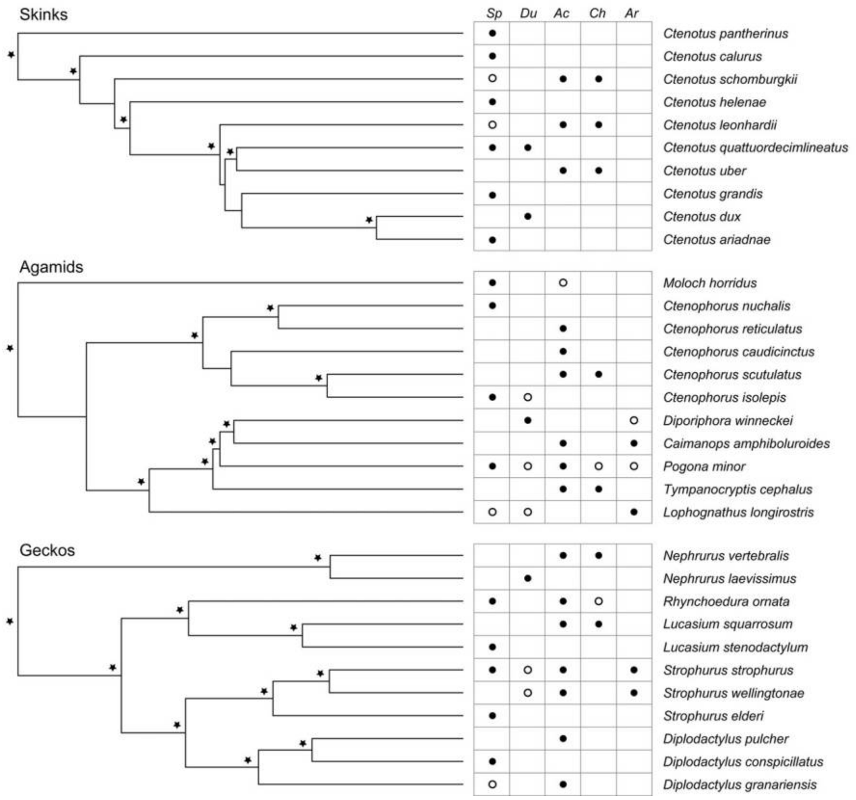

Figure 3: Phylogenetic relationships and macrohabitat use for *Ctenotus* skinks, agamids, and diplodactyline geckos from the Lorna Glen study region. Branch lengths are in relative time units, and all trees are scaled to a common basal divergence of 1.0 time units before present. Asterisks denote nodes with posterior probabilities greater than 0.95. Filled and open circles denote, respectively, strong and weak affinities of each taxon for a given habitat category: Sp, spinifex sandplain; Du, dune; Ac, *Acacia* woodland; Ch, chenopod shrubs on hard soils; Ar, arboreal.

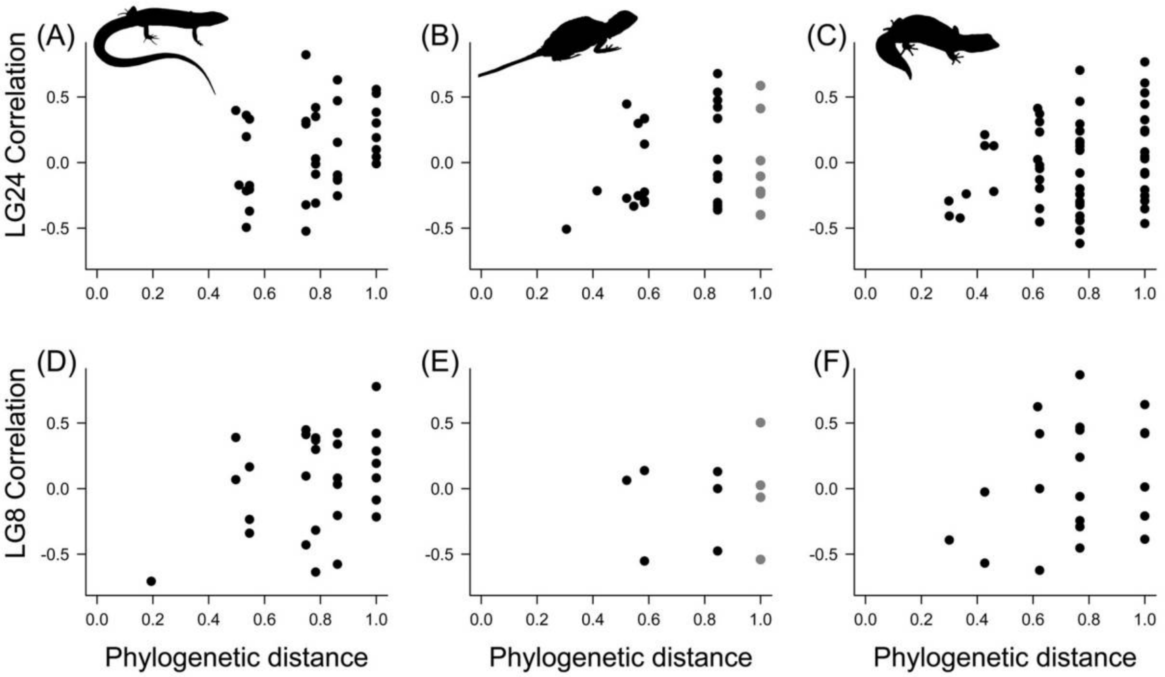

Figure 4: Pairwise correlations in species abundance across all sites in the LG24 (*top*) and LG8 (*bottom*) data sets as a function of phylogenetic distance for three clades. *A*, *D*, *Ctenotus* skinks; *B*, *E*, agamids; *C*, *F*, diplodactyline geckos. Positive relationships imply that closely related species show greater contrasts in abundance and occurrence across sites than more distantly related species. Significant positive correlations are observed for both skinks and geckos for both data sets (table 1) and reflect phylogenetic evenness. The relationship is significant for agamids in the LG24 data set after points that include *Moloch horridus* (gray circles) are removed. Phylogenetic distances are on relative timescales and cannot be compared among clades.

explain the phylogenetic evenness observed under the simple Mantel test; closely related species are found together less often than expected even after explicitly species-specific habitat preferences are accounted for. In contrast, there is no evidence that closely related agamid species show contrasting patterns of abundance ($P \geq .54$ for all analyses) under a null model that assumes habitat filtering. This suggests that, for agamids, a tendency for closely related species to have divergent habitat preferences can explain the pattern of phylogenetic evenness observed when *Moloch* is excluded from the analysis.

Ecological Niche Evolution

Five PC axes explained 96% of the variation in habitat use among species (table B1, available online). However, PC1 accounted for more than 59% of this variation, with PC2 and other axes explaining less than 15% each. Species from each clade are highly dispersed in multivariate habitat space corresponding to PC1 and PC2 (fig. 5). Striking differences in habitat use among the three focal clades are immediately apparent. For example, five of the 10 *Ctenotus* species in the Lorna Glen region are essentially specialists on spinifex sandplain habitats (fig. 5*B*: PC1 > 0, PC2 < 0), whereas comparatively few species of agamids and geckos utilize this habitat. In contrast, no Lorna Glen *Ctenotus* species utilize rocky habitats with extensive tree cover, whereas multiple species of both agamids and geckos can be found in these habitats (e.g., PC1 < 0; PC2 < 0).

We found no evidence that closely related species from any clade were more similar in habitat use than expected under a simple randomization test ($K < 0.9$ for all clades and PC axes; table B2, available online). Rather, K statistics for the dominant PC1 axis for both agamids and geckos were lower than expected (agamids: $K = 0.54$, $P = .001$; geckos: $K = 0.46$, $P = .060$), indicating a negative relationship between phylogenetic relatedness and similarity in habitat use. This implies either that closely related species are excessively divergent or that convergence has occurred among distantly related species within these two clades. Visual inspection of species positions on PC1 and PC2 axes suggests substantial divergence in habitat attributes among the most closely related species pairs, particularly for agamids and geckos (fig. 5; closely related species

Table 1: Correlations between pairwise phylogenetic distances and pairwise rank correlations in abundance for three lizard clades

Data set, clade	N	Correlation	Mantel P value	EC P value
LG24:				
Skinks	9	.292	.048	.001
Agamids	9	.137	.324	.740
Agamids (no *Moloch*)	8	.258	.024	.538
Geckos	11	.23	.029	.036
LG8:				
Skinks	8	.342	.096	.001
Agamids	5	−.044	.694	.740
Agamids (no *Moloch*)	4	.097	.98	.94
Geckos	7	.303	.344	.016

Note: The P values (two-tailed) denote the probability of observing absolute correlations greater than or equal to the observed value under the null model. Significance was assessed by means of Mantel matrix permutations or an environmentally constrained (EC) model that incorporated habitat filtering into the simulation of null communities. N is the number of species in each data set. Agamids (no *Moloch*) refers to analyses conducted after the species *Moloch horridus* was excluded from the data set. N for each data set is less than the total regional richness (fig. 3) because some species were unique to either the LG24 or the LG8 data set.

pairs are denoted by identically colored [nonblack] points). Closely related species pairs tend to be highly differentiated on PC1, separating cleanly on an ecological axis of hard/rocky soils versus sandy substrates. *Ctenotus quattuordecimlineatus*, for example, is found in open dune habitats with loose sand substrate, whereas *Ctenotus uber* occurs on hard, stony substrates dominated by chenopod shrubs. However, this is not a general rule. The most closely related pair of *Ctenotus* species, *Ctenotus dux* and *Ctenotus ariadnae*, is poorly differentiated on PC1 but well separated on PC2. Although both species use sandy substrates, *C. dux* uses open dune habitats, whereas *C. ariadnae* is found in sandplains with heavy cover of mature spinifex.

We tested whether divergence in habitat use between such closely related pairs was greater than expected under a Brownian motion model of trait evolution. Euclidean distances between species pairs computed from the first five PC axes trend toward ecological divergence; closely related species pairs from all three clades are at least weakly divergent relative to the null expectation ($P = .05$–$.07$; table 2). These results provide quantitative validation of the visual impression that such species pairs are well separated in the multivariate state space defined by the PC1 and PC2 axes (fig. 5).

Patterns of dietary divergence with respect to phylogeny contrast sharply with patterns observed for habitat use (fig. 6). In *Ctenotus*, there is no relationship between dietary overlap and phylogenetic distance ($r = 0.280$, $P = .396$, two-tailed). However, dietary overlap is negatively correlated with phylogenetic distance for both agamids ($r = -0.504$, $P = .048$) and geckos ($r = -0.414$, $P = .068$). The result for agamids is even more striking when the highly specialized *Moloch* is excluded from the analysis ($r = -0.667$, $P = .034$). In *Ctenotus*, the observed lack of relationship between phylogeny and dietary divergence does not result from exceptional lability of dietary attributes, and diets are highly conserved. Mean pairwise diet overlap for Lorna Glen *Ctenotus* species is 0.92, much higher than that between *Ctenotus* species and members of the other clades (mean *Ctenotus*-agamid overlap = 0.37; mean *Ctenotus*-gecko overlap = 0.56). Thus, in contrast to patterns of habitat use, desert lizard diets are phylogenetically conserved, with more distantly related species pairs of agamids and geckos showing progressively greater divergence in prey species utilization.

Discussion

We explored ecological and phylogenetic community structure in species from three major evolutionary radiations that are represented by high regional and local diversity in Australia's Great Victoria Desert. Superficially, skinks, agamids, and geckos are characterized by similar patterns of phylogenetic community structure. All three clades showed evidence of phylogenetic evenness (table 1; fig. 3): closely related pairs of species show low co-occurrence across sites relative to more divergent species pairs. These patterns are consistent with those observed in some previous studies of vertebrates at similar spatial scales (Lovette and Hochachka 2006; Cooper et al. 2008), although Gomez et al. (2010) documented a shift from evenness to clustering in Amazonian antbird communities at fine spatial scales. Phylogenetic evenness could, in prin-

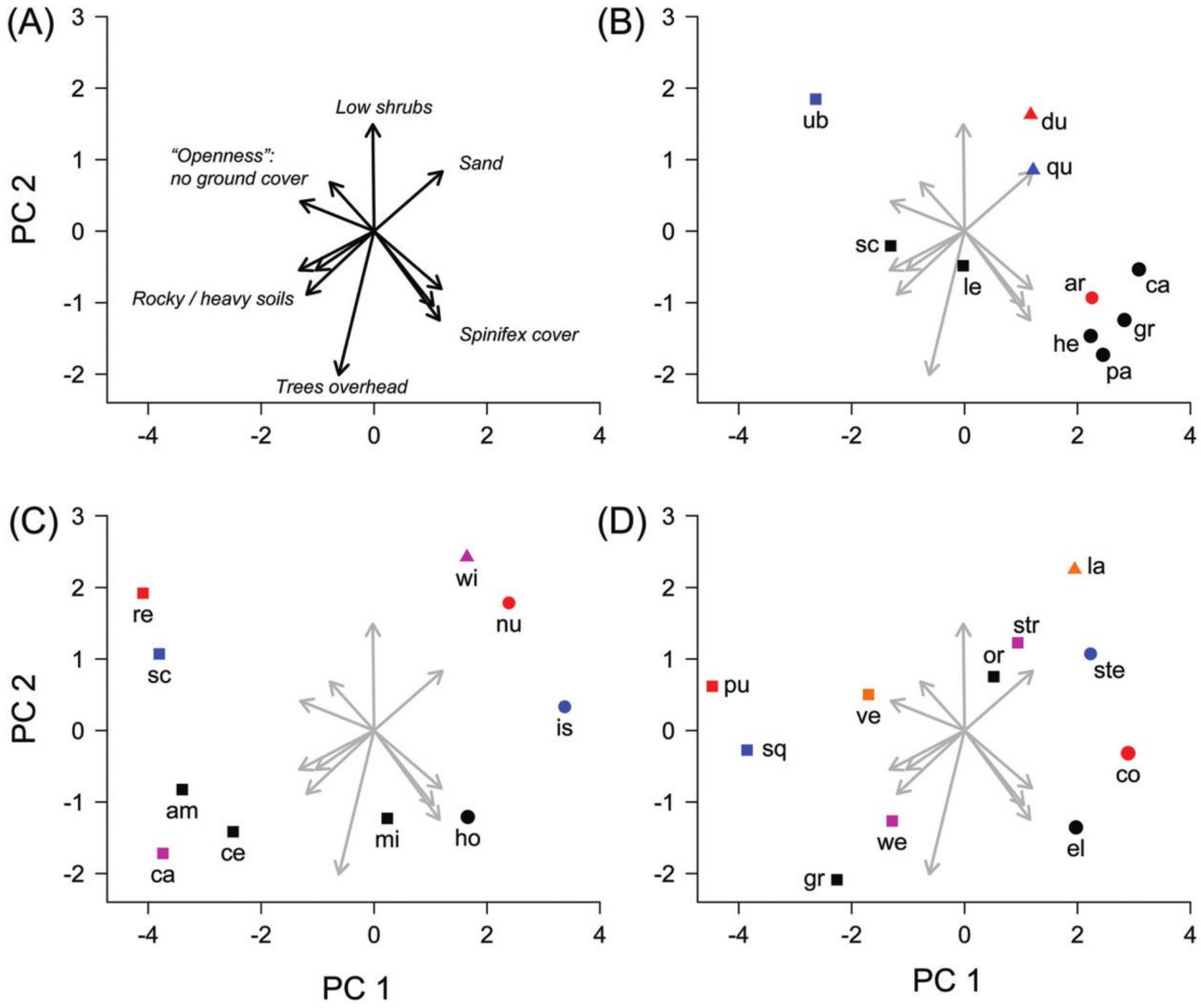

Figure 5: Position of Lorna Glen lizard species in multivariate habitat space. *A*, Relative loadings and interpretation of habitat variables on principal-component (PC) axes 1 and 2. *B–D*, Species scores on PC1 and PC2 for *Ctenotus* skinks (*B*), agamids (*C*), and geckos (*D*). Abbreviations correspond to the first two letters of each species' name. Points corresponding to closely related species pairs (fig. 3; table 2) use identical (nonblack) colors. Circles and triangles denote sandplain and dune specialists, respectively; squares denote other habitat associations and/or generalists. Closely related species pairs are characterized by highly divergent patterns of habitat use.

ciple, result from either competitive exclusion or habitat filtering (Webb et al. 2002; Cavender-Bares et al. 2009), depending on the phylogenetic distribution of species' habitat attributes. To distinguish between these possibilities, we used an environmentally constrained null model that explicitly accounted for habitat filtering; patterns of evenness that remain significant under this model are likely to reflect contemporary ecological interactions between species rather than divergence or convergence in habitat use.

After patterns of habitat use are accounted for, our results suggest that the ecological mechanisms leading to phylogenetic evenness are not identical for the focal clades. For agamids, phylogenetic evenness can likely be attributed to highly divergent patterns of habitat use among closely related species pairs (fig. 5*C*). When null communities are generated by assigning species to sites on the basis of habitat suitability, we find no evidence for phylogenetic evenness (table 1). This suggests that divergent patterns of habitat use among closely related species account for the observed tendency toward evenness when the highly divergent *Moloch* is removed from the analysis. Consistent with this possibility, we found support for exceptional divergence in habitat use among closely related pairs of aga-

Table 2: Euclidean distances in principal-component (PC) scores for all monophyletic species pairs within the Lorna Glen study region, relative to values expected under a model of trait evolution by Brownian motion

Clade, species pair	Abbreviation	Distance (*Z* score)	*P*
Skink			.068
Ctenotus ariadnae, *Ctenotus dux*	ar, du	3.2 (.99)	
Ctenotus quattuordecimlineatus, *Ctenotus uber*	qu, ub	6.4 (1.59)	
Agamid			.050
Ctenophorus isolepis, *Ctenophorus scutulatus*	is, sc	7.6 (1.85)	
Diporiphora winneckei, *Caimanops amphiboluroides*	wi, am	6.69 (.86)	
Ctenophorus nuchalis, *Ctenophorus reticulatus*	nu, re	5.71 (.69)	
Gecko			.052
Nephurus vertebralis, *Nephurus laevissimus*	ve, la	4 (.60)	
Strophurus wellingtonae, *Strophurus strophurus*	we, st	3.5 (.33)	
Diplodactylus pulcher, *Diplodactylus conspicillatus*	pu, co	7.3 (1.67)	
Lucasium stenodactylum, *Lucasium squarrosum*	st, sq	6.5 (1.30)	

Note: The *Z* value is the standardized effect size corresponding to the observed distance; positive *Z* values indicate that observed distances are greater than expected under a model of trait evolution by Brownian motion. Species pairs include all monophyletic pairs of species within the regional species pool (fig. 3). Taxon abbreviations (e.g., ar, du) correspond to the first two letters of the specific name and are given for reference to figure 5. Euclidean distances were calculated between species scores on the first five PC axes. The *P* values (two-tailed) were computed for each clade by combining *Z* scores across species pairs using the unweighted *Z*-transform (Whitlock 2005).

mid species ($K = 0.54$; table 2). For example, *Ctenophorus scutulatus* and *Ctenophorus isolepis* are more closely related than any other agamids in the region, yet they are highly divergent in ecology. *Ctenophorus scutulatus* inhabits *Acacia* woodlands and chenopod shrub–dominated habitats on hard, stony soils, whereas *C. isolepis* occurs primarily in sandplain and dune habitats. Although both of these species are abundant and widespread in the study region, they were never recorded at the same site.

In contrast, phylogenetic evenness persists for both *Ctenotus* skinks and geckos after accounting for habitat filtering (table 1). Species assemblages at any particular site form a phylogenetically overdispersed subset of species, relative to those that would be predicted based on species' habitat preferences alone. Closely related species of geckos are exceptionally divergent in patterns of habitat use ($K = 0.46$; fig. 5; table 2), implying that both historical divergence in species' habitat preferences and contemporary species interactions may influence the distribution of individuals across the Lorna Glen landscape. For *Ctenotus*, the observed *K* statistic is not significantly different from the randomized expectation (table A5, available online), but the two most closely related species pairs are divergent (fig. 5).

These results suggest that contemporary ecological interactions limit the co-occurrence of close relatives and are inconsistent with "community drift" within particular habitat types, whereby species undergo constrained (strict neutrality; Hubbell 2001) or unconstrained fluctuations in abundance. Such non-niche-based models of community assembly should lead to random phylogenetic structure within local communities (Webb et al. 2002; Kraft et al. 2007) or even clustering if phylogenetic signal in metacommunity abundance is present (Kembel 2009). Likewise, if habitat-associated niche differences are not important for species coexistence, we should not observe phylogenetically overdispersed patterns of habitat use. Rather, we would expect species' habitat use to be clustered or random with respect to phylogeny, depending on the tempo and mode of trait evolution through time (Revell et al. 2008; Ackerly 2009); this is clearly not what we observe for skinks, geckos, or agamids. Distributions of body size in local communities of *Ctenotus* skinks are also consistent with an important role for current or historical species interactions. Rabosky et al. (2007*a*) found that co-occurring sets of species showed greater divergence in body size than would be expected several null models of community assembly.

One apparent contradictory result is that phylogenetic evenness in geckos and skinks persists after accounting for habitat filtering but closely related species in both of these clades are more divergent in habitat use than expected. One possible explanation is that the environmentally constrained (EC) null model fails to fully account for major axes of habitat divergence between species. If species are similar in most habitat attributes but differ sharply in several attributes that have great effect on observed site abundance patterns, then this dynamic may not have been captured in the EC null model but could have been captured by analyses of habitat niche evolution. However, we do not believe that these results are necessarily contradictory. Although the most closely related species generally appear to be exceptionally divergent in habitat use (table 2), species pairs of intermediate relatedness that utilize broadly

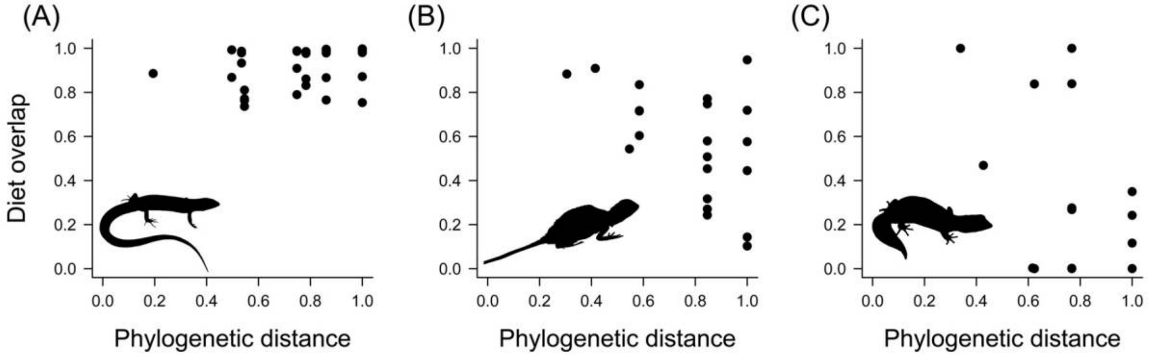

Figure 6: Pairwise dietary overlap as a function of phylogenetic distance for skinks (*A*), agamids (*B*), and geckos (*C*). *Ctenotus* skinks are characterized by high dietary overlap, and the magnitude of overlap is uncorrelated with phylogenetic distance ($r = 0.28$; two-tailed $P = .40$). However, more distantly related pairs of geckos and agamids show decreasing dietary overlap (agamids: $r = -0.50$, $P = .048$; geckos: $r = -0.41$, $P = .068$). Dietary overlap within agamid and gecko clades is low compared to that in *Ctenotus* skinks.

similar habitat types may co-occur less than expected because of local competitive interactions. For example, *Ctenotus quattuordecimlineatus* and *Ctenotus dux* are moderately close relatives (fig. 3). Although both species utilize dune habitats, they were never captured at the same site.

Our results for dietary niche evolution provide a striking contrast to those obtained for habitat use. Closely related species pairs in all three clades are characterized by phylogenetic conservatism of prey utilization (fig. 6). However, similarity in diet decays rapidly with increasing phylogenetic distance for both agamids and geckos, and the most distantly related gecko species pairs show very low dietary overlap. *Ctenotus* represents a radiation much younger than that of either the geckos or the agamids we studied: the basal divergence among the Lorna Glen species may have occurred as recently as 12 Ma, compared to 20–25 Ma for agamids and at least 30 Ma for diplodactyline geckos (Rabosky et al. 2007*b*; Hugall et al. 2008; Oliver and Sanders 2009). It is possible that *Ctenotus* species have simply not had sufficient time to evolve distinct differences in utilization of major prey categories. Although we focused on within-clade patterns of dietary niche evolution, our results mirror those for squamate reptiles at much larger phylogenetic scales (Vitt et al. 1999; Vitt and Pianka 2005). Conservatism of squamate diets at the scale of "major clades" may reflect constraints on the evolution of chemosensory and prey capture mechanisms (Vitt et al. 2003), but we do not yet understand why similar patterns might be present at the much finer phylogenetic scales we consider here.

Evolutionary Paths of Least Resistance

One possible explanation for the contrasting results we observe for diet and habitat is that divergence along at least one major niche axis is necessary for species coexistence. Thus, the intensity of competition (or potential for competition) along one niche axis, such as diet, might favor divergence along another axis (habitat) to minimize competitive interactions. Our findings are broadly similar to patterns of "niche complementarity" observed in communities of *Anolis* lizards, whereby ecological similarity along a given niche axis is negatively correlated with divergence along other niche axes (Losos et al. 2003). Such a pattern has been noted for North America wood warblers, where distantly related species that co-occur tend to differ in foraging mode (Lovette and Hochachka 2006). This model contrasts somewhat with the "α early, β throughout" model of Ackerly et al. (2006), who found that for some radiations, traits associated with local coexistence tended to diverge early during evolutionary radiations, whereas habitat-associated traits diverged throughout.

Previous studies in a range of vertebrate taxa have found habitat use to be an extremely labile trait (Richman and Price 1992; Bohning-Gaese and Oberrath 1999; Vitt et al. 1999; Bohning-Gaese et al. 2003; Ackerly et al. 2006). In light of the well-documented conservatism of squamate reptile diets, we suggest that divergence in habitat might represent an evolutionary path of least resistance (Schluter 1996). However, while habitat use is evolutionarily labile for many groups, there is no general consensus on the relative conservatism and lability of vertebrate diets at the phylogenetic scales considered in this study. For example, trophic guild shows at least moderate phylogenetic conservatism in Lake Tanganyika cichlids (Wagner et al. 2009), but *Sebastes* rockfish show low phylogenetic signal in trophic position (Ingram 2011). Our results also provide an intriguing contrast to many examples of diversification

in postglacial lakes, where habitat and diet are strongly coupled and frequently undergo concerted shifts. For example, species from multiple clades of fishes have undergone intralacustrine divergence into benthic and limnetic habitat specialists, with concomitant changes in prey utilization (Malmquist et al. 1992; Bernatchez et al. 1999; Schluter 2000).

Convergence, Divergence, and the Legacy of Interspecific Competition

Patterns of phylogenetic evenness within local communities are typically interpreted as consistent with two processes: (1) competitive exclusion of closely related (and ecologically similar) species and (2) environmental filtering on convergently evolved traits (Webb et al. 2002; Cavender-Bares et al. 2004; Kraft et al. 2007; Emerson and Gillespie 2008). We have demonstrated that similarity in habitat use is negatively associated with phylogenetic distance for geckos, agamids, and skinks (e.g., $K \ll 1$). At least in part, it is possible that environmental filtering on convergent traits can explain the observed patterns. Closely related species may thus appear to be exceptionally divergent because more distantly related taxa have converged on similar patterns of habitat use.

However, it is also possible that the observed divergence in habitat use reflects a tendency for closely related species to evolve distinct habitat preferences. This would be expected if species interactions have led to character displacement in habitat-associated traits (Schluter and McPhail 1992; Robinson and Wilson 1994; Pfennig and Pfennig 2009) or if speciation itself is sometimes associated with divergence in habitat (Diamond 1986). This latter possibility may be especially relevant for Australian agamids, which are characterized by temporally declining rates of speciation and trait evolution suggestive of "niche-filling" models of diversification (Harmon et al. 2003; Rabosky and Lovette 2008). There is no reason why greater dissimilarity among closely related species necessarily implies convergence of distantly related taxa, and this widespread assumption should be avoided in the absence of formal tests for convergent evolution.

Such patterns need not entail character displacement or convergent evolution but can arise as a consequence of filtering processes that occur at the level of the regional species pool. For example, suppose that traits associated with habitat use show an absence of phylogenetic signal (Richman and Price 1992; Losos et al. 2003), perhaps because of limits on the total amount of trait space that can be explored (Revell et al. 2008). If other traits important in resource use show phylogenetic signal or conservatism (Losos 2008), then competitive interactions may ensure that only species that have diverged greatly in habitat can occur within the same regional species pool. In this scenario, species interactions do not directly mediate divergence in habitat-associated traits but nonetheless shape the distribution of traits within regional pools (Colwell and Winkler 1984).

We suggest that negative phylogeny-habitat correlations within regional species pools are most likely to reflect the historical legacy of interspecific competition, or "the ghost of competition past" (Connell 1980). It is clear that divergent character displacement and regional pool filtering (Colwell and Winkler 1984; Brown et al. 2000) are processes that involve a direct role for interspecific competition, and both could lead to patterns similar to those we observed in the three focal radiations. However, interspecific competition can also influence patterns of convergence among taxa. If environmental filtering on convergent traits leads to patterns of phylogenetic evenness (Webb et al. 2002; Cavender-Bares et al. 2004; Kraft et al. 2007), then it must be the case that close relatives have divergent habitat preferences. Were this not the case, they would occur in the same habitat or local community, and phylogenetic clustering would be observed. This is potentially problematic, because we must explain why distantly related taxa, but not closely related taxa, converge on similar patterns of habitat use. The evolutionary and ecological mechanisms by which this might occur remain poorly explored, but one possible explanation is that the potential for competition between closely related species prevents them from converging on similar patterns of habitat use. Thus, the relevant question is not necessarily why distantly related species show convergent traits but why closely related species would have failed to converge.

Conclusions

Much previous work has debated the evolutionary and ecological mechanisms that underlie the exceptional lizard species richness of arid Australia (Pianka 1972, 1989; Morton and James 1988; James 1991; James and Shine 2000; Daly et al. 2007; Rabosky et al. 2007*b*). Our results suggest an important role for species interactions in mediating both divergence in habitat between species and species co-occurrence patterns within major habitat classes. These patterns are inconsistent with a prominent role for ecological community drift and with other processes that fail to posit a central role for interspecific competition, such as random evolutionary divergence of species' niche parameters.

Our results do not imply that species richness is decoupled from regional and/or historical biogeographic processes (Ricklefs 1987; Cornell and Lawton 1992), and some evidence suggests that species richness in the Australian deserts may be shaped by the overall size of the

arid zone (James and Shine 2000) as well as by diversification patterns of constituent clades (Rabosky et al. 2007*b*). An exciting frontier in this and other systems is to understand how evolutionary processes of speciation and extinction can interact with geographic area to generate differences in local community richness, even when local community structure appears to be strongly influenced by species interactions.

Acknowledgments

We thank C. Dardia, A. Davis Rabosky, S. Donnellan, P. Doughty, J. Huelsenbeck, M. Hutchinson, C. Kovach, A. McCune, and L. Stenzler for logistical and other assistance. We especially thank E. Pianka; this study would not have been possible without his pioneering work on arid-zone lizard ecology. I. Kealley, as Goldfields Regional Manager of the Department of Environment and Conservation, was instrumental in providing resources and long-term support for fieldwork at Lorna Glen. S. Campbell provided valuable support on many surveys and coordinated site establishment, and Conservation Volunteers Australia assisted with site installation. Numerous individuals contributed to field surveys, including B. Barton, B. Gardiner, G. Hearle, R. How, J. and J. Kavanagh, B. Lewis, R. Lynch, D. Mason, J. Patten, A. Richardson, P. Spencer, L. Stokes, P. J. Waddell, and A. Woosnam. We thank J. Cavender-Bares and two anonymous reviewers for comments that greatly improved the manuscript. This research was supported by National Science Foundation (NSF) grants OSIE-0612855 and DEB-0814277, the Goldfields Region of the Western Australia Department of Environment and Conservation, the Fuller Evolutionary Biology Program at the Cornell Laboratory of Ornithology, and the Miller Institute for Basic Research in Science at the University of California, Berkeley. D.L.R.'s field sampling at Lorna Glen was conducted under permit SF0004654 (Western Australia Department of Environment and Conservation).

Literature Cited

Ackerly, D. 2009. Conservatism and diversification of plant functional traits: evolutionary rates versus phylogenetic signal. Proceedings of the National Academy of Sciences of the USA 106:19699–19706.

Ackerly, D., and M. J. Donoghue. 1998. Leaf size, sapling allometry, and Corner's rules: phylogeny and correlated evolution in maples (*Acer*). American Naturalist 152:767–791.

Ackerly, D. D., D. W. Schwilk, and C. O. Webb. 2006. Niche evolution and adaptive radiation: testing the order of trait divergence. Ecology 87:S50–S61.

Adams, D. C., and J. O. Church. 2008. Amphibians do not follow Bergmann's rule. Evolution 62:413–420.

Bernatchez, L., A. Chouinard, and G. Q. Lu. 1999. Integrating molecular genetics and ecology in studies of adaptive radiation: whitefish, *Coregonus* sp., as a case study. Biological Journal of the Linnean Society 68:173–194.

Blomberg, S. P., T. Garland Jr., and A. R. Ives. 2003. Testing for phylogenetic signal in comparative data: behavioral traits are more labile. Evolution 57:717–745.

Bohning-Gaese, K., and R. Oberrath. 1999. Phylogenetic effects on morphological, life-history, behavioural and ecological traits of birds. Evolutionary Ecology Research 1:347–364.

Bohning-Gaese, K., M. D. Schuda, and A. J. Helbig. 2003. Weak phylogenetic effects on ecological niches of Sylvia warblers. Journal of Evolutionary Biology 16:956–965.

Brandley, M. C., A. Schmitz, and T. W. Reeder. 2005. Partitioned Bayesian analyses, partition choice, and the phylogenetic relationships of scincid lizards. Systematic Biology 54:373–390.

Brown, J. H., B. J. Fox, and D. A. Kelt. 2000. Assembly rules: desert rodent communities are structured at scales from local to continental. American Naturalist 156:314–321.

Brown, J. H., E. J. Bedrick, S. K. M. Ernest, J.-L. E. Cartron, and J. F. Kelly. 2004. Constraints on negative relationships: mathematical causes and ecological consequences. Pages 298–307 *in* M. L. Taper and S. Lele, eds. The nature of scientific evidence. University of Chicago Press, Chicago.

Cadotte, M. W., T. J. Davies, J. Regetz, S. W. Kembel, E. Cleland, and T. H. Oakley. 2009. Phylogenetic diversity metrics for ecological communities: integrating species richness, abundance and evolutionary history. Ecology Letters 13:96–105.

Cavender-Bares, J., D. D. Ackerly, D. A. Baum, and F. A. Bazzaz. 2004. Phylogenetic overdispersion in Floridian oak communities. American Naturalist 163:823–843.

Cavender-Bares, J., K. H. Kozak, P. V. A. Fine, and S. W. Kembel. 2009. The merging of community ecology and phylogenetic biology. Ecology Letters 12:693–715.

Colwell, R. K., and D. W. Winkler. 1984. A null model for null models in biogeography. Pages 344–359 *in* D. R. Strong, D. Simberloff, L. G. Abele, and A. B. Thistle, eds. Ecological communities: conceptual issues and the evidence. Princeton University Press, Princeton, NJ.

Connell, J. H. 1980. Diversity and the coevolution of competitors, or the ghost of competition past. Oikos 35:131–138.

Cooper, N., J. Rodriguez, and A. Purvis. 2008. A common tendency for phylogenetic overdispersion in mammalian assemblages. Proceedings of the Royal Society B: Biological Sciences 275:2031–2037.

Cornell, H. V., and J. H. Lawton. 1992. Species interactions, local and regional processes, and limits to the richness of ecological communities: a theoretical perspective. Journal of Animal Ecology 61:1–12.

Daly, B. G., C. R. Dickman, and M. S. Crowther. 2007. Selection of habitat components by two species of agamid lizards in sandridge desert, central Australia. Austral Ecology 32:825–833.

———. 2008. Causes of habitat divergence in two species of agamid lizards in arid central Australia. Ecology 89:65–76.

Darwin, C. 1859. On the origin of species by means of natural selection, or the preservation of favoured races in the struggle for life. J. Murray, London.

Diamond, J. M. 1986. Evolution of ecological segregation in the New Guinea montane avifauna. Pages 98–125 *in* J. M. Diamond and T. J. Case, eds. Community ecology. Harper & Row, New York.

Drummond, A. J., and A. Rambaut. 2007. BEAST: Bayesian evolutionary analysis sampling trees. BMC Evolutionary Biology 7:214.

Drummond, A. J., S. W. Y. Ho, M. J. Phillips, and A. Rambaut. 2006. Relaxed phylogenetics and dating with confidence. PLoS Biology 4(5):e88.

Edwards, S. V., L. Liu, and D. K. Pearl. 2007. High-resolution species trees without concatenation. Proceedings of the National Academy of Sciences of the USA 104:5936–5941.

Emerson, B. C., and R. G. Gillespie. 2008. Phylogenetic analysis of community assembly and structure over space and time. Trends in Ecology & Evolution 23:619–630.

Environment Australia. 2000. Revision of the interim biogeographic regionalisation for Australia (IBRA) and development of version 5.1. Environment Australia, Canberra.

Gomez, J. P., G. A. Bravo, R. T. Brumfield, J. G. Tello, and C. D. Cadena. 2010. A phylogenetic approach to disentangling the role of competition and habitat filtering in community assembly of Neotropical forest birds. Journal of Animal Ecology 79:1181–1192.

Gotelli, N. J. 2000. Null model analysis of species co-occurrence patterns. Ecology 81:2606–2621.

Harmon, L. J., and R. E. Glor. 2010. Poor statistical performance of the Mantel test in phylogenetic comparative analyses. Evolution 64:2173–2178.

Harmon, L. J., J. A. Schulte, A. Larson, and J. B. Losos. 2003. Tempo and mode of evolutionary radiation in iguanian lizards. Science 301:961–964.

Harmon, L. J., J. T. Weir, C. Brock, R. E. Glor, and W. E. Challenger. 2008. GEIGER: investigating evolutionary radiations. Bioinformatics 24:129–131.

Helmus, M. R., K. Savage, M. W. Diebel, J. T. Maxted, and A. R. Ives. 2007. Separating the determinants of phylogenetic community structure. Ecology Letters 10:917–925.

Hubbell, S. P. 2001. The unified neutral theory of biodiversity and biogeography. Princeton University Press, Princeton, NJ.

Hugall, A., R. Foster, M. N. Hutchinson, and M. S. Y. Lee. 2008. Phylogeny of Australasian agamid lizards based on nuclear and mitochondrial genes: implications for morphological evolution and biogeography. Biological Journal of the Linnean Society 93: 343–358.

Ingram, T. 2011. Speciation along a depth gradient in a marine adaptive radiation. Proceedings of the Royal Society B: Biological Sciences 278:613–618.

Ives, A. R., and M. R. Helmus. 2010. Phylogenetic metrics of community similarity. American Naturalist 176:E128–E142.

James, C. D. 1991. Temporal variation in diets and trophic partitioning by coexisting lizards (*Ctenotus*, Scincidae) in central Australia. Oecologia (Berlin) 85:553–561.

James, C. D., and R. Shine. 2000. Why are there so many coexisting species of lizards in Australian deserts? Oecologia (Berlin) 125: 127–141.

Keane, T. M., C. J. Creevey, M. M. Pentony, T. J. Naughton, and J. O. McInerney. 2006. Assessment of methods for amino acid matrix selection and their use on empirical data shows that ad hoc assumptions for choice of matrix are not justified. BMC Evolutionary Biology 6:29.

Kembel, S. W. 2009. Disentangling niche and neutral influences on community assembly: assessing the performance of community phylogenetic structure tests. Ecology Letters 12:949–960.

Kembel, S. W., P. D. Cowan, M. R. Helmus, W. K. Cornwell, H. Morlon, D. D. Ackerly, S. P. Blomberg, and C. O. Webb. 2010. Picante: R tools for integrating phylogenies and ecology. Bioinformatics 26:1463–1464.

Kraft, N. J. B., W. K. Cornwell, C. O. Webb, and D. D. Ackerly. 2007. Trait evolution, community assembly, and the phylogenetic structure of ecological communities. American Naturalist 170:271–283.

Leaché, A. D. 2009. Species tree discordance traces to phylogeographic clade boundaries in North American fence lizards (*Sceloporus*). Systematic Biology 58:547–559.

Leaché, A. D., and B. Rannala. 2010. The accuracy of species tree estimation under simulation: a comparison of methods. Systematic Biology 60:126–137.

Liu, L. 2008. BEST: Bayesian estimation of species trees under the coalescent model. Bioinformatics 24:2542–2543.

Liu, L., and D. K. Pearl. 2007. Species trees from gene trees: reconstructing Bayesian posterior distributions of a species phylogeny using estimated gene tree distributions. Systematic Biology 56:504–514.

Losos, J. B. 2008. Phylogenetic niche conservatism, phylogenetic signal and the relationship between phylogenetic relatedness and ecological similarity among species. Ecology Letters 11:995–1003.

Losos, J. B., M. Leal, R. E. Glor, K. de Queiroz, P. E. Hertz, L. R. Schettino, A. C. Lara, T. R. Jackman, and A. Larson. 2003. Niche lability in the evolution of a Caribbean lizard community. Nature 424:542–545.

Lovette, I. J., and W. M. Hochachka. 2006. Simultaneous effects of phylogenetic niche conservatism and competition on avian community structure. Ecology 87(suppl.):S14–S28.

Malmquist, H. J., S. S. Snorrason, S. Skulason, B. Jonsson, O. Sandlund, and P. M. Jonasson. 1992. Diet differentiation in polymorphic arctic charr in Thingvallavatn, Iceland. Journal of Animal Ecology 61:21–35.

Mayfield, M., and J. Levine. 2010. Opposing effects of competitive exclusion on the phylogenetic structure of communities. Ecology Letters 13:1085–1093.

Melville, J., J. A. Schulte, and A. Larson. 2001. A molecular phylogenetic study of ecological diversification in the Australian lizard genus *Ctenophorus*. Journal of Experimental Zoology 291:339–353.

———. 2004. A molecular study of phylogenetic relationships and evolution of antipredator strategies in Australian *Diplodactylus geckos*, subgenus *Strophurus*. Biological Journal of the Linnean Society 82:123–138.

Melville, J., L. J. Harmon, and J. B. Losos. 2006. Intercontinental community convergence of ecology and morphology in desert lizards. Proceedings of the Royal Society B: Biological Sciences 273: 557–563.

Morton, S. R., and C. D. James. 1988. The diversity and abundance of lizards in arid Australia: a new hypothesis. American Naturalist 132:237–256.

Nylander, J. A. A., J. C. Wilgenbusch, D. L. Warren, and D. L. Swofford. 2008. AWTY (are we there yet?): a system for graphical exploration of MCMC convergence in Bayesian phylogenetics. Bioinformatics 24:581–583.

Oksanen, J., F. G. Blanchet, R. Kindt, P. Legendre, P. R. Minchin, R. G. O'Hara, G. L. Simpson, P. Solymos, M. H. H. Stevens, and H. Wagner. 2010. vegan: community ecology package. R package, version 1.17-0. http://CRAN.R-project.org/package=vegan.

Oliver, P. M., and K. L. Sanders. 2009. Molecular evidence for Gondwanan origins of multiple lineages within a diverse Australasian gecko radiation. Journal of Biogeography 36:2044–2055.

Peres-Neto, P. R. 2004. Patterns in the co-occurrence of fish species in streams: the role of site suitability, morphology and phylogeny versus species interactions. Oecologia (Berlin) 140:352–360.

Peres-Neto, P. R., J. D. Olden, and D. A. Jackson. 2001. Environmentally constrained null models: site suitability as occupancy criterion. Oikos 93:110–120.

Pfennig, K. S., and D. W. Pfennig. 2009. Character displacement: ecological and reproductive responses to a common evolutionary problem. Quarterly Review of Biology 84:253–276.

Pianka, E. R. 1969*a*. Habitat specificity, speciation, and species density in Australian desert lizards. Ecology 50:498–502.

———. 1969*b*. Sympatry of desert lizards (*Ctenotus*) in western Australia. Ecology 50:1012–1030.

———. 1972. Zoogeography and speciation of Australian desert lizards. Copeia 1972:127–145.

———. 1973. The structure of lizard communities. Annual Review of Ecology and Systematics 4:53–74.

———. 1986. Ecology and natural history of desert lizards. Princeton University Press, Princeton, NJ.

———. 1989. Desert lizard diversity: additional comments and some data. American Naturalist 134:344–364.

Pianka, E. R., and H. D. Pianka. 1970. Ecology of *Moloch horridus* (Lacertilia: Agamidae) in western Australia. Copeia 1970:90–103.

Rabosky, D. L., and I. J. Lovette. 2008. Explosive evolutionary radiations: decreasing speciation or increasing extinction through time? Evolution 62:1866–1875.

Rabosky, D. L., J. Reid, M. A. Cowan, and J. Foulkes. 2007*a*. Community-wide overdispersion of body size in Australian desert lizard communities. Oecologia (Berlin) 154:561–570.

Rabosky, D. L., S. C. Donnellan, A. L. Talaba, and I. J. Lovette. 2007*b*. Exceptional among-lineage variation in diversification rates during the radiation of Australia's most diverse vertebrate clade. Proceedings of the Royal Society B: Biological Sciences 274:2915–2923.

Rabosky, D. L., A. L. Talaba, S. C. Donnellan, and I. J. Lovette. 2009. Molecular evidence for hybridizaton between two Australian desert skinks, *Ctenotus leonhardii* and *Ctenotus quattuordecimlineatus* (Scincidae: Squamata). Molecular Phylogenetics and Evolution 53: 368–377.

Read, J. L. 1995. Subhabitat variability: a key to the high reptile diversity in chenopod shrublands. Australian Journal of Ecology 20:494–501.

Revell, L. J., L. J. Harmon, and D. C. Collar. 2008. Phylogenetic signal, evolutionary process, and rate. Systematic Biology 57:591–601.

Richman, A. D., and T. Price. 1992. Evolution of ecological differences in the Old World leaf warblers. Nature 355:817–821.

Ricklefs, R. E. 1987. Community diversity: relative roles of local and regional processes. Science 235:167–171.

———. 2011. Applying a regional community concept to forest birds of eastern North America. Proceedings of the National Academy of Sciences of the USA 108:2300–2305.

Robinson, B. W., and D. S. Wilson. 1994. Character release and displacement in fishes: a neglected literature. American Naturalist 144:596–627.

Sanderson, M. J., D. Boss, D. Chen, K. A. Cranston, and A. Wehe. 2008. The PhyLoTA browser: processing GenBank for molecular phylogenetics research. Systematic Biology 57:335–346.

Schluter, D. 1996. Adaptive radiation along genetic lines of least resistance. Evolution 50:1766–1774.

———. 2000. The ecology of adaptive radiation. Oxford University Press, Oxford.

Schluter, D., and J. D. McPhail. 1992. Ecological character displacement and speciation in sticklebacks. American Naturalist 140:85–108.

Seaman, D. E., and R. A. Powell. 1996. An evaluation of the accuracy of kernel density estimators for home range analysis. Ecology 77: 2075–2085.

Skinner, A., A. F. Hugall, and M. N. Hutchinson. 2011. Lygosomine phylogeny and the origins of Australian scincid lizards. Journal of Biogeography 38:1044–1058.

Ulrich, W., and N. J. Gotelli. 2010. Null model analysis of species associations using abundance data. Ecology 91:3384–3397.

Vamosi, S. M., S. B. Heard, J. C. Vamosi, and C. O. Webb. 2009. Emerging patterns in the comparative analysis of phylogenetic community structure. Molecular Ecology 18:572–592.

Vidal, N., and S. B. Hedges. 2009. The molecular evolutionary tree of lizards, snakes, and amphisbaenians. Comptes Rendus Biologies 332:129–139.

Vitt, L. J., and E. R. Pianka. 2005. Deep history impacts present-day ecology and biodiversity. Proceedings of the National Academy of Sciences of the USA 102:7877–7881.

Vitt, L. J., P. A. Zani, and M. C. Esposito. 1999. Historical ecology of Amazonian lizards: implications for community ecology. Oikos 87:286–294.

Vitt, L. J., E. R. Pianka, W. E. Cooper, and K. Schwenk. 2003. History and the global ecology of squamate reptiles. American Naturalist 162:44–60.

Wagner, C. E., P. B. McIntyre, K. S. Buels, D. M. Gilbert, and E. Michel. 2009. Diet predicts intestine length in Lake Tanganyika's cichlid fishes. Functional Ecology 23:1122–1131.

Webb, C. O. 2000. Exploring the phylogenetic structure of ecological communities: an example for rain forest trees. American Naturalist 156:145–155.

Webb, C. O., D. D. Ackerly, M. A. McPeek, and M. J. Donoghue. 2002. Phylogenies and community ecology. Annual Review of Ecology and Systematics 33:475–505.

Whitlock, M. C. 2005. Combining probability from independent tests: the weighted *Z*-method is superior to Fisher's approach. Journal of Evolutionary Biology 18:1368–1373.

Associate Editor: Stephen B. Heard
Editor: Mark A. McPeek
